# Identification of Molecular Pathways Facilitating Glioma Cell Invasion *In Situ*

**DOI:** 10.1371/journal.pone.0111783

**Published:** 2014-11-03

**Authors:** Ido Nevo, Kevin Woolard, Maggie Cam, Aiguo Li, Joshua D. Webster, Yuri Kotliarov, Hong Sug Kim, Susie Ahn, Jennifer Walling, Svetlana Kotliarova, Galina Belova, Hua Song, Rolanda Bailey, Wei Zhang, Howard A. Fine

**Affiliations:** 1 Neuro-Oncology Branch, National Cancer Institute, National Institute of Neurological Disorders and Stroke, National Institutes of Health, Bethesda, Maryland, United States of America; 2 Laboratory of Cancer Biology and Genetics, National Cancer Institute, National Institutes of Health, Bethesda, Maryland, United States of America; University of Alabama at Birmingham, United States of America

## Abstract

Gliomas are mostly incurable secondary to their diffuse infiltrative nature. Thus, specific therapeutic targeting of invasive glioma cells is an attractive concept. As cells exit the tumor mass and infiltrate brain parenchyma, they closely interact with a changing micro-environmental landscape that sustains tumor cell invasion.

In this study, we used a unique microarray profiling approach on a human glioma stem cell (GSC) xenograft model to explore gene expression changes *in situ* in Invading Glioma Cells (IGCs) compared to tumor core, as well as changes in host cells residing within the infiltrated microenvironment relative to the unaffected cortex. IGCs were found to have reduced expression of genes within the extracellular matrix compartment, and genes involved in cell adhesion, cell polarity and epithelial to mesenchymal transition (EMT) processes. The infiltrated microenvironment showed activation of wound repair and tissue remodeling networks. We confirmed by protein analysis the downregulation of EMT and polarity related genes such as *CD44* and *PARD3* in IGCs, and *EFNB3*, a tissue-remodeling agent enriched at the infiltrated microenvironment. *OLIG2*, a proliferation regulator and glioma progenitor cell marker upregulated in IGCs was found to function in enhancing migration and stemness of GSCs. Overall, our results unveiled a more comprehensive picture of the complex and dynamic cell autonomous and tumor-host interactive pathways of glioma invasion than has been previously demonstrated. This suggests targeting of multiple pathways at the junction of invading tumor and microenvironment as a viable option for glioma therapy.

## Introduction

Glioblastoma (GBM), the most malignant glioma tumor, is highly invasive with a median survival of 16 months despite combined surgical resection, radiotherapy, and chemotherapy. Although the molecular signature of glioma has been investigated in detail [1]–[3], these studies have focused on the primary tumor and not on the invading cells and their microenvironment. By nature of their unique phenotype it is reasonable to assume that the invading cells in GBM have gene regulatory networks that differ from noninvasive cells within the primary tumor bulk. The present study was specifically aimed at identifying molecular pathways responsible for and potential therapeutic targets against glioma invasion.

The brain consists of numerous cell types forming tissue scaffolds that guide invasion [4]. Glioma cell invasion into brain tissue occurs along preexisting myelinated fibers and blood vessels, similar to the natural migratory patterns of neural stem cells (NSC) and progenitor cells [5]. It is clearly a multifactorial process that employs simultaneous interactions of invading glioma cells (IGCs) with the surrounding components of the tumor microenvironment. Further characterization of these cellular compartments may help in the development of novel strategies to therapeutically inhibit glioma invasion and progression.

Glioma stem cells (GSCs) are a subpopulation of cells in the tumor capable of self-renewal that can give rise to a heterogeneous tumor [6]. In xenograft models, GSCs extensively infiltrate into the surrounding cerebral cortex, phenocopying a pathognomonic feature of human GBM [7].

Ideally, one would perform between–group differential expression analysis for each cellular compartment. However, experimental methods for isolating the IGCs and their microenvironment cellular compartment are neither timely nor cost effective, and isolating them *in vitro* would affect cell physiology and gene expression. Traditional laser capture microdissection studies of glioma invasion focused on glioma specimens from human patients, dissecting neoplastic astrocytes as the invading cells at the rim of the tumor core [8], [9]. These studies, however did not evaluate the more deeply invasive glioma cells nor did they interrogate the contribution of host cells residing within the invaded cerebral cortex.

In the present study we used an innovative laser microdissection (LMD) enrichment strategy and gene expression analysis. Differentially expressed genes (DEGs) of IGCs and their microenvironment were simultaneously detected allowing us to deduce functional networks that characterize glioma invasion *in situ*. Subsequently, we found several genes in multiple pathways that may facilitate invasion which were validated at the protein level in xenografted GSCs and in clinical samples from GBM patients. Importantly, *OLIG2*, a proliferation regulator and glioma progenitor cell marker was upregulated in the IGCs relative to the tumor core. Inhibition of *OLIG2* expression reduced migration and stemness and provided functional confirmation as a potential therapeutic target in glioma invasion. Overall, the novel approach in this study allowed us to construct and evaluate a more complete picture of multiple pathways within the area of glioma invasion than has been previously demonstrated.

## Material and Methods

Following signed informed consent, tumor tissue was obtained from patients (aged ≥18 years) undergoing medically indicated resection of malignant gliomas at the National Institutes of Health as part of a clinical trial approved by the Institutional Review Board (NCI-02C0140). NCI Animal Care and Use Committee (ACUC) approved all animal experiments.

### GSC Cultures

Primary glioma stem cells were cultured in NBE medium as previously described [10]. 0923 and 1228A1 GSC lines (previously derived from patient samples following the approval of National Cancer Institute Institutional Review Board [7], [10]) were used for intracranial mouse glioma models.

### Real-Time Measurement of Cell Migration

GSCs migration was assessed by the xCELLigence RTCA DP device, according to the manufacturer instructions (Roche Diagnostics, Mannheim, Germany).

### Intracranial Tumor Mouse Model, LMD, and Gene Expression Microarray

An intracranial orthotopic model in SCID mice was utilized for the generation of infiltrative glioma xenograft tumors [7]. NCI animal use and care committee approved all animal experiments. Brains were handled in RNase free conditions, imbedded in OCT compound (Sakura Finetek, CA), frozen immediately and kept at −80°C. Serial sections (10 µm) were mounted on MembraneSlides and processed for LMD using Leica LMD6000 (Leica Microsystems). Total cellular RNA isolation was carried out for Affymetrix HG-U133 plus2 or Mouse430_2 GeneChip Arrays, according to the manufacturer instructions (Affymetrix, CA).

Raw data for this experiment has been deposited in the Gene Expression Omnibus (accession number GSE53717).

### NanoString Gene Expression Quantification

The probe for each gene was designed and synthesized by NanoString nCounter technologies to match the Affymetrix probe target region (Table S1). Housekeeping genes picked based on the gene-expression data were added to the CodeSet. Probes were designed with no cross-species (human-mouse) hybridization. Quantification analysis of the mRNA transcript was done according to the manufacturer's recommendations using 65 ng of total RNA for each LMD area, from four injected mice of each xenografted GSC line. Data were analyzed using the nCounter digital analyzer software using the human and mouse housekeeping genes.

### Immunohistochemistry and Immunoblots

The standard immunohistochemistry of paraffin sections and western blotting were performed as previously described [7]. A veterinarian pathologist examined histological staining and distinguished invading human glioma cells from other cells by nuclear size or human-nuclei staining (Figure S3). Images were captured using a Zeiss LSM 510 confocal microscope.

## Results

### GSCs Migration *In Vitro* and *In Vivo*

**Figure 1:**
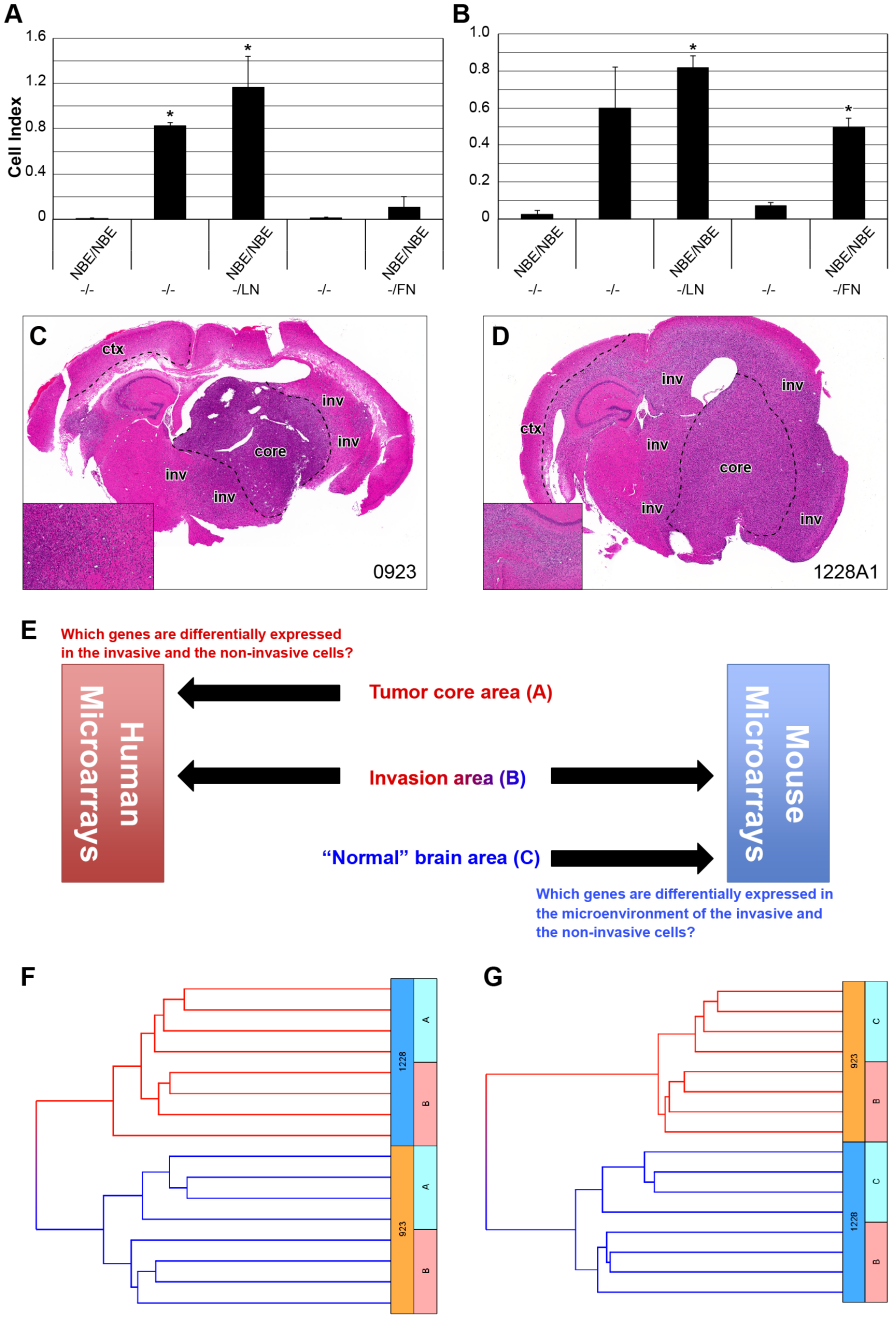
GSCs migration, scheme of experiment and hierarchal clustering. Migration activity of (A) 0923 and (B) 1228A1 GSCs. The bars represent the average cell indices at 13h for the indicated conditions of at least 3 experiments. Asterisks indicate significant (p≤0.01) differences in the migration of the cells compared to the control [NBE medium in both upper and lower chambers (NBE/NBE), uncoated well (−/−)] as determined by *t*-test. Error bars indicate SEM. The migratory nature of xenografted (C) 0923 and (D) 1228A1 GSCs. H&E stained sections depicting diffuse infiltration of GBM cells. Inset depicts at 10× magnification, a representative infiltrative area. Regions of LMD are indicated: tumor core (core), area of invasion (inv) and normal cortex tissue (ctx) on the sections. (E) Samples at the AOI (region B) were hybridized to human gene expression arrays, followed by second hybridization to mouse expression arrays. Tumor core (Region A) and “normal” brain area (region C) samples were hybridized to human and mouse gene expression arrays, respectively. Unsupervised hierarchal clustering shows segregation in the human arrays between the tumor core (region A) and the AOI (region B) (F). Clean separation was also observed in the mouse arrays between the “normal” brain and the AOI (G).

GSCs derived from primary GBM show a predilection for migrating along white matter tracts such as the corpus callosum, as is characteristically seen in patient brains [7]. Thus, we first evaluated the migration capability of 0923 and 1228A1 GSCs using an *in vitro* cell migration assay (Figures 1A and 1B). Both GSC lines demonstrated significant migration capability in haptotaxis conditions with laminin compared to cells in control uncoated wells (*P*≤0.01). When laminin was used for chemotaxis, only 0923 cells showed significant migration (*P*≤0.01). In haptotaxis conditions with fibronectin, only 1228A1 cells demonstrated significant migration (*P*≤0.01). None of the GSCs migrated in chemotaxis with fibronectin. Intracranial tumors generated by 0923 and 1228A1 GSCs demonstrated an extensive infiltration into the surrounding cerebral cortex (Figures 1C and 1D). In contrast to these GSC lines, 0827A2 GSCs showed limited infiltration capability *in vivo*, although they demonstrated significant migration capability *in vitro* (Figures S1A and S1B). We therefore explored the cell autonomous and microenvironmental molecular signals responsible for glioma invasion on 0923 and 1228A1 GSCs xenografts.

### Gene Expression of Invasive Glioma Cells and Their Microenvironment

To identify genes associated with glioma cell invasion and the tumor microenvironment, we utilized xenografts of 0923 and 1228A1 GSCs, which displayed an invasive phenotype *in vivo* (Figure 1C and 1D). We investigated whether there are DEGs between cells in the tumor core and IGCs, as well as between the microenvironment at the area of invasion (AOI) and normal brain tissue distant from the tumor. Tissue samples were collected using LMD from three distinct regions of each injected mouse: tumor core area (region A), infiltration enriched region (region B, AOI) and “normal” brain area (region C, reactive mouse cells distant from the tumor). RNA from each region was isolated and subjected to a whole-genome expression array analysis. As depicted in Figure 1E, samples of region B were hybridized on human gene expression arrays, followed by a second hybridization on mouse expression arrays. Region A and C samples were hybridized to human and mouse gene expression arrays, respectively.

Applying the algorithm described in the supporting information section (File S1), we identified 756 human DEGs in the tumor core and the area of invasion, and 1566 mouse DEGs in the “normal” brain area and the AOI, common to both xenografted GSC lines. These differentially expressed gene sets were then designated as the glioma dataset and the microenvironment dataset, correspondingly. Hierarchical clustering of the DEGs in each dataset segregated the arrays according to the GSC lines. Moreover, a clear separation was noticed between the human arrays of the tumor core and the area of invasion (Figure 1F). Similarly, the invasive and the “normal” areas of the mouse brain were segregated (Figure 1G).

### GO Enrichment of Genes Involved in Glioma Invasion

**Figure 2:**
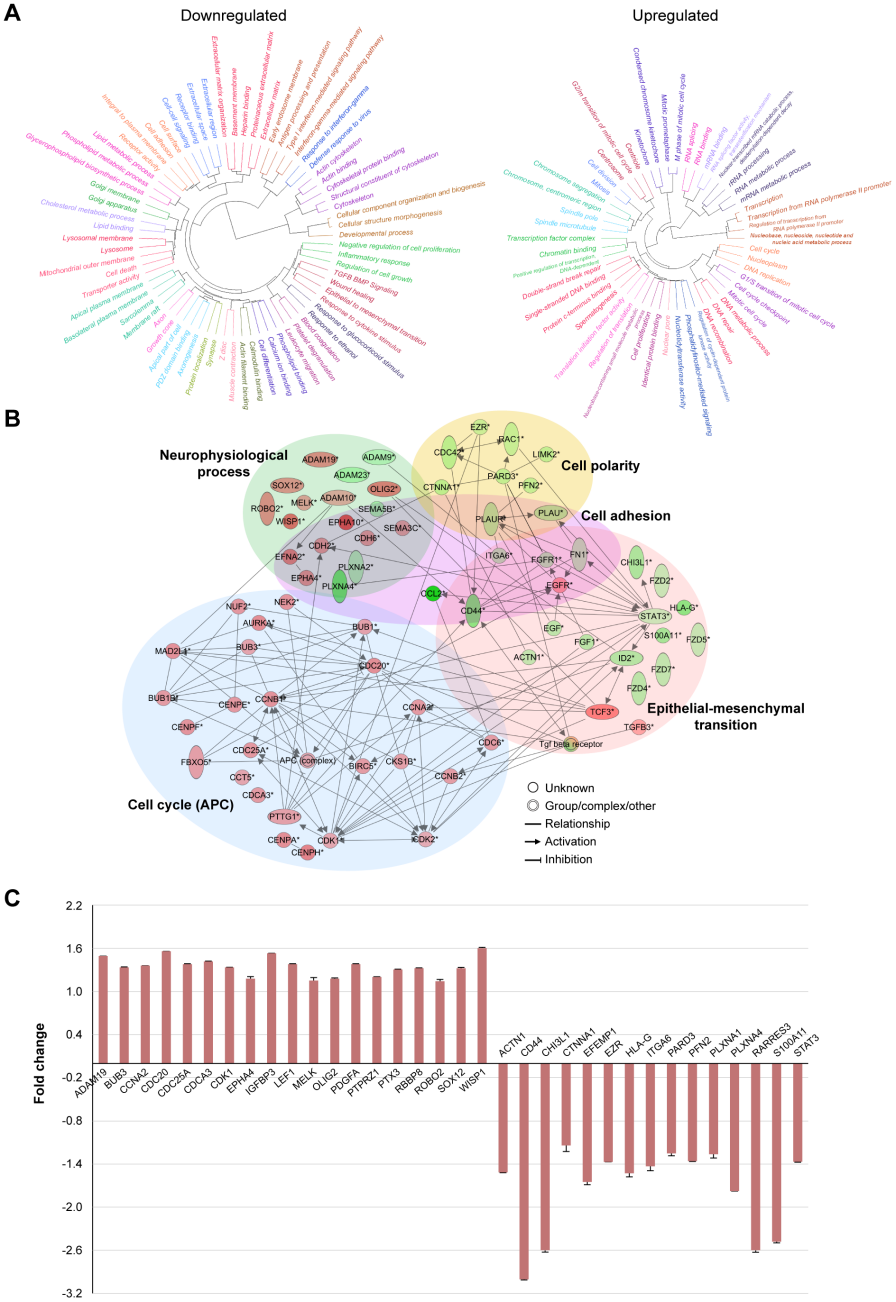
Gene ontology and pathway analysis of invading GSCs. (A) GO analysis of the IGCs dataset. The GO terms were clustered by category gene memberships and significantly overrepresented (*P*≤0.05) in up- and downregulated genes and had at least a 30% overlap with at least one other category. (B) Global network of invading GSCs was generated using IPA. Five major functional networks are associated with IGCs. Red and green nodes indicate up- and down-regulated genes, respectively. (C) Differentially expressed genes in IGCs validated by NanoString.

Up- and down-regulated genes in each dataset were analyzed by Gene Ontology (GO) enrichment analysis. GO terms that were overrepresented and had at least a 30% overlap with at least one other category were identified as significant (*P*≤0.05) in glioma invasion (Figure 2A and Figure S2A). As shown in Figure 2A, many of the enriched GO terms were associated with tumor cell invasion, most notably downregulated genes that were mapped to cell adhesion, extracellular space, cell-cell signaling, epithelial to mesenchymal transition (EMT), and actin cytoskeleton; while the upregulated genes were mapped to GO terms associated with transcription, cell cycle checkpoint and mitotic transitions.

In the microenvironment dataset (Figure S2A) downregulated genes were enriched in categories involved in neurophysiological processes such as potassium ion transport, synaptic transmission, and neuron projection development. Intriguingly, upregulated genes were enriched in processes involving viral host interaction, apoptosis process, as well as antigen processing and presentation of peptide antigen via MHC class I, possibly an inflammatory response to the tumor cells. In addition, upregulated genes mapped to nitrogen compound metabolic process may imply oxidative stress resulting from glia-mediated inflammation.

### Functional Networks in Glioma Invasion

For every dataset, we focused on a subset of genes associated with significant cellular processes and canonical pathways (Table S2 in File S1) that were identified by the MetaCore database. Other genes from the datasets were supplemented based on functional association as defined by literature references. To investigate the interconnection of genes expressed in the IGCs with other gene products, pathways, and biological processes, a global molecular network was formed using the Ingenuity Pathway Analysis (IPA) software. Five major networks were identified: EMT, cell polarity, neurophysiological process, cell cycle (anaphase promoting complex, APC) and cell adhesion (Figure 2B). Interestingly, 16 out of 20 genes involved in EMT (e.g. *STAT3*, *CHI3L1*, *CD44*) and 9 genes of cell polarity (e.g. *PARD3*, *CDC42*, *PLAUR*) were downregulated [2], [11]–[14]. *STAT3*, which is downregulated, acts as a hub in the EMT network interacting with all other networks. On the other hand, majority of genes (13 out of 18) associated with neurophysiological process (e.g. *OLIG2*, *WISP1*, *EPHA4*, *PLXNA4*) and in the cell cycle (e.g. *APC complex*, *CDC20*, *FBXO5*) were upregulated [15]–[20]. Figure 2B shows how these genes and networks relate to one another. These genes are related to key biological functions such as cell cycle, cellular growth and proliferation and cellular movement (data not shown). Similar analysis of the microenvironment dataset revealed a major network with direct and indirect functional relationships between gene products (Figure S2B). Beta-catenin (*CTNNB1*), which plays a central role in the canonical Wnt pathway, acts as a major hub interconnecting with genes and gene products associated with tissue remodeling and wound repair (e.g. *PLAU*, *CDH2*, *NCAM1*, *BMP7*, *TGFB3*) [21]–[25].

Further analysis identified 17 potential protein-protein binding interactions between the IGCs and the microenvironment residing host cells (Table S3 in File S1). Six out of the 17 interactions have been previously linked to invasion, migration or adhesion (*EPHA4-Efnb3*, *SEMA5A-Plxnb3*, *AQP4-Dag1*, *FGFR1-Ncam1*, *FGF2-Sdc2*, and *APP-Aplp2*) [20], [26]–[29] whereas only 3 interactions have been previously reported in glioma (*SEMA5A-Plxnb3*, *AQP4-Dag1*, and *FGF2-Sdc2*) [20], [26]–[31].

### Validation of Invasion-Related DEGs in GSC Xenograft Samples

Validation of DEGs at the transcript level was performed using NanoString for 34 human and 23 mouse genes of interest, using the same RNA samples used for microarray (Figures 2C and S2C). We prioritized genes from the functional networks that showed a significant change (p<0.05) in both xenografted GSC lines while few were selected based on biological interest. Our data show strong concordance of fold-change and p-value between the NanoString and the microarray data (Tables S4 and S5 in File S1).

**Figure 3:**
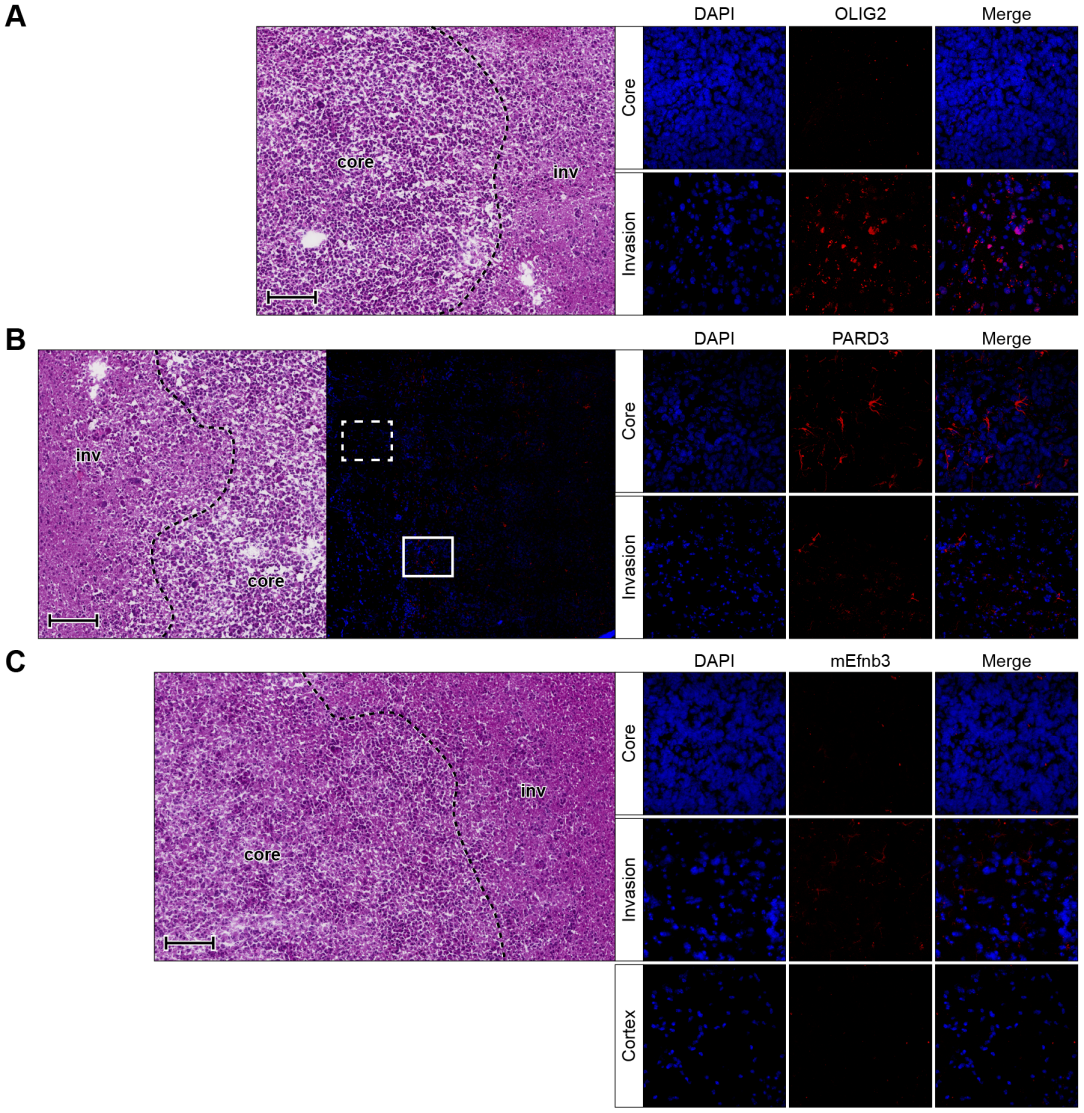
*OLIG2*, *PARD3* and *Efnb3* expression in IGCs of xenografted 0923 GSCs. Frozen sections of xengraft glioma derived from intracranial injection of 0923 GSCs were stained with (A) *OLIG2*, (B) *PARD3* or (C) *Efnb3* (all in red). DNA was stained with 4′,6-diamidino-2-phenylindole (DAPI; blue). At the left side of each panel: intracranial tumor histology (H&E, scale bar, 100 µm) and when available a whole brain tile at same scale. Solid line box (tumor core) and dashed line box (AOI) identify magnified (×40) images on right as indicated.

**Figure 4:**
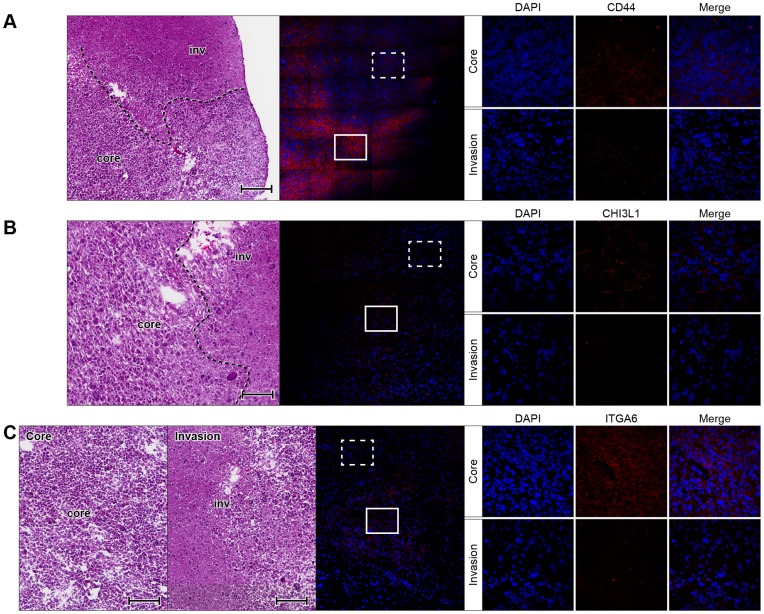
Downregulated expression of EMT associated genes in IGCs of xenografted 0923 GSCs. Frozen sections of xenograft glioma derived from intracranial injection of 0923 GSCs were stained with (A) *CD44*, (B) *CHI3L1* or (C) *ITGA6* (all in red). Please see legend of Figure 3.

These DEGs were further confirmed at the protein level using immunofluorescence labeling on brain tissue frozen sections of the xenografted GSCs. As shown in Figure 3A and Figure S4A, *OLIG2* expression is present in the nuclei of IGCs, while no expression was detected in the tumor core. Human *PARD3* expression was detected in the plasma membrane of cells at the tumor core but not at the AOI (Figure 3B and Figure S4B). In addition, a potential cell-cell interaction between *Efnb3* expressing cells at the microenvironment and *EPHA4* expressing glioma cells at the AOI was suggested (Table S3 in File S1). Efnb3 was detected in the plasma membrane of mouse cells at the AOI (Figure 3C and Figure S4C) but not in the unaffected cortex or the tumor core, while human *EPHA4* expression was found in the plasma membrane of the IGCs (Figure S5A). The expression of *CD44*, *CHI3L1* and *ITGA6* are all associated with EMT [2], which were scarcely detected in the AOI as compared to their elevated expression at the tumor core (Figure 4 and Figure S6).

We further investigated the protein expression of several other genes. The elevated expression of *WISP1* (*CCN4*) and *PDGFA* proteins in the invasive glioma cells was confirmed (Figures S5B and S7A). Interestingly, cell-surface expression of m*Pdgfra* was only detected in host cells residing at the AOI (Figure S7B) and the sonic hedgehog (*Shh*) protein was only observed at the brain cortex and barely detected at the AOI or tumor core (Figure S5C). These proteins play an important role in cancer biology by paracrine and autocrine effects on tumor cells and host cells residing at the tumor microenvironment [32]–[34]. Altogether, the immunohistochemistry results confirmed our initial gene-expression data and NanoString validation.

### Validation of Invasion-Related DEGs in Patient Samples

**Figure 5:**
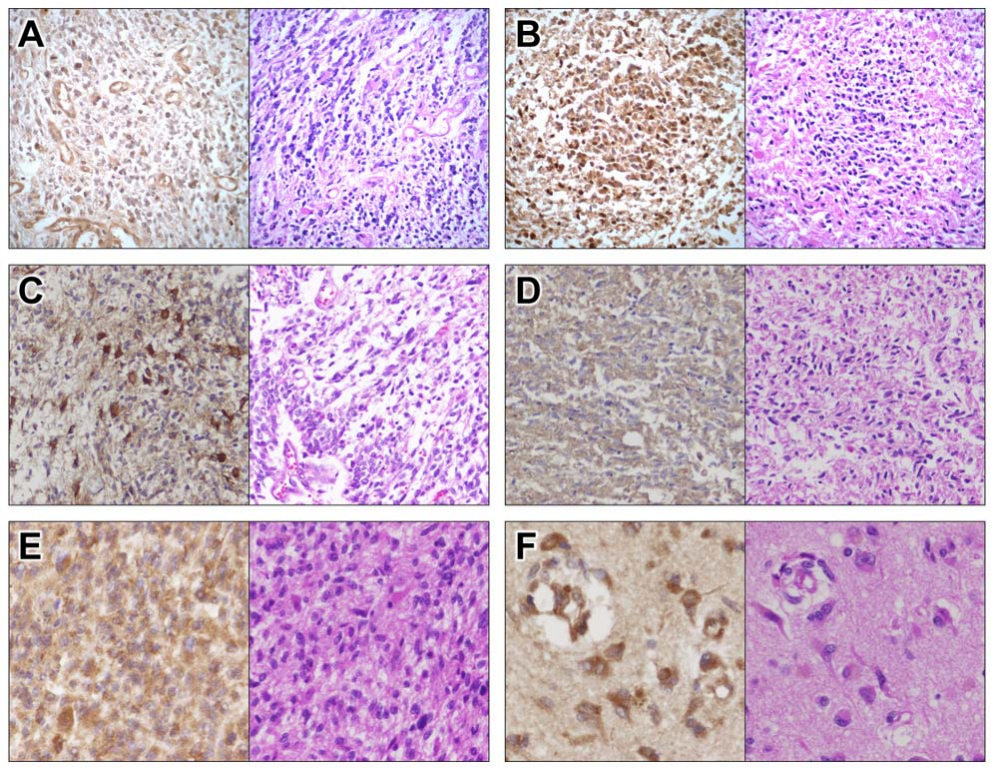
*OLIG2*, *PARD3* and *EFNB3* expression in GBM patients. Immunohistochemistry and H&E staining of matched tumor core (A, C, E) and AOI (B, D, F) specimens from GBM patients. Representative images for *OLIG2* upregulation (A, B) and decreased expression of *PARD3* (C, D) in the IGCs. *EFNB3* expression (E, F) is enriched at the AOI. Magnification ×40.

The clinical relevance of our data was tested by immunohistochemistry of paired tumor core and invasive sections of FFPE tissue from GBM patients. Figure 5 shows significant expression of *OLIG2* in cell nuclei and reduced expression of *PARD3* in plasma membrane in the AOI compared to the tumor core. *EFNB3* was enriched in the plasma membrane and cytoplasm of host cells with an astrocytic appearance that reside within the AOI. The staining for *OLIG2*, *PARD3* and *EFNB3* showed marked heterogeneity between patients in terms of both intensity and distribution, ranging from a few weakly positive cells to apparent overexpression.

### Therapeutic Significance of the Inhibition of *OLIG2* Expression

**Figure 6:**
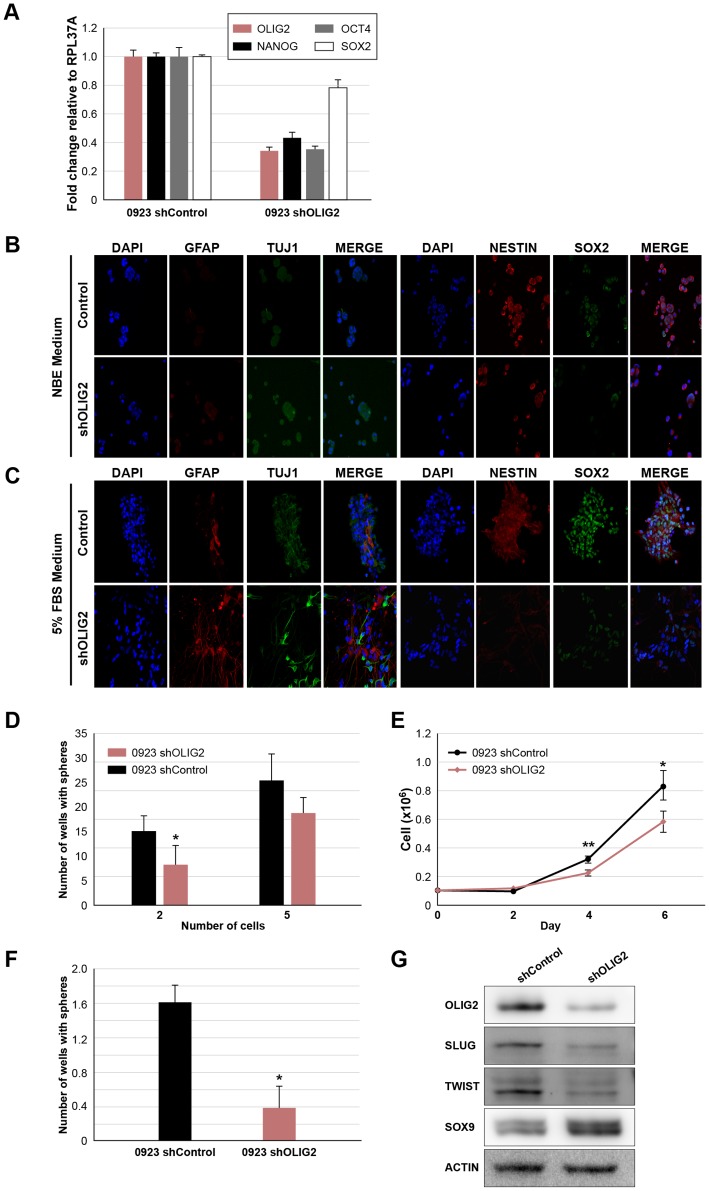
Therapeutic potential of the inhibition of *OLIG2* expression. (A) Quantitative RT-PCR of *OLIG2*, *NANOG*, *OCT4* and *SOX2*. Graph indicates mRNA fold change relative to *RPL37A* mRNA. Error bars represent SD (performed in triplicates, p≤0.01 as determined by *t*-test). 0923 shOLIG2 and shControl GSCs, were cultured in NBE medium (B) or 5% FBS differentiation medium (C) and immunostained for *NESTIN*, *SOX2*, *GFAP* and *TUJ1* as indicated (Magnification ×20). shOLIG2 *vs.* shControl 0923 GSCs self-renewal and proliferation examined by limiting dilution (D) and cell counting (E) assays (*t*-test, *p≤0.05, **p≤0.01). Error bars indicate SD of at least 3 independent experiments. (F) Migration of shOLIG2 compared to shControl 0923 GSCs. The bars represent the average cell indices at 13 h for the indicated conditions of at least 3 experiments (p≤0.05 as determined by *t*-test). Error bars indicate SEM. (G) Western blot analysis of shOLIG2 and shControl 0923 GSCs with antibodies against *OLIG2* and central regulators of EMT during neural crest cell migration and cancer: *SLUG*, *TWIST* and *SOX9*. *ACTIN* was used as loading control.

*OLIG2*, a basic helix-loop-helix (bHLH) transcription factor, is selectively expressed in a subgroup of glioma cells and required for glioma formation in a murine GBM model [35]. *OLIG*2 expression by the IGCs *in situ* may suggest their stemness, which supports their invasive capability *in vivo*. We thus investigated whether *OLIG2* could serve as a therapeutic target in invading glioma cells. We examined the effect of *OLIG2* knockdown (shOLIG2) on the differentiation state, self-renewal, proliferation, cell migration and *in vivo* invasion of GSCs. As demonstrated in Figure 6A, the mRNA expression levels of the NSC markers: *NANOG*, *OCT4* and *SOX2* in 0923 shOLIG2 GSCs were significantly decreased compared to the shControl cells. Immunofluorescence labeling (Figure 6B) also confirmed the significant decrease of *SOX2* and *NESTIN* expression in 0923 shOLIG2 GSCs. Culturing the cells in medium containing serum abolished the expression of these markers in 0923 shOLIG2 cells (Figure 6C). Unlike normal NSCs, GSCs weakly co-stained for both *GFAP* and *TUJ1* (markers for glial and neuronal lineages, respectively) as previously demonstrated [7]. Serum-containing medium increased the expression of *GFAP* and *TUJ1* in 0923 shOLIG2 cells in a more pronounced manner than in shControl cells (Figure 6C). Next we preformed limiting dilution and proliferation assays to determine the effect of shOLIG2 on self-renewal and cell growth. 0923 shOLIG2 cells exhibit relatively poor self-renewal capability (Figure 6D) and reduced cell proliferation by 30% (Figure 6E). Intriguingly, the migration of 0923 shOLIG2 GSCs was decreased by 75% (Figure 6F). We further demonstrated the involvement of EMT in glioma invasion and tested by western blot analysis the effect of shOLIG2 on the expression of central regulators of EMT during neural crest cell migration and cancer: *TWIST*, *SNAI2* (*SLUG*) and *SOX9*. Our analysis revealed (Figure 6G) that 0923 shOLIG2 GSCs exhibit reduced levels of *SNAI2* and *TWIST* and elevated *SOX9* levels.

**Figure 7:**
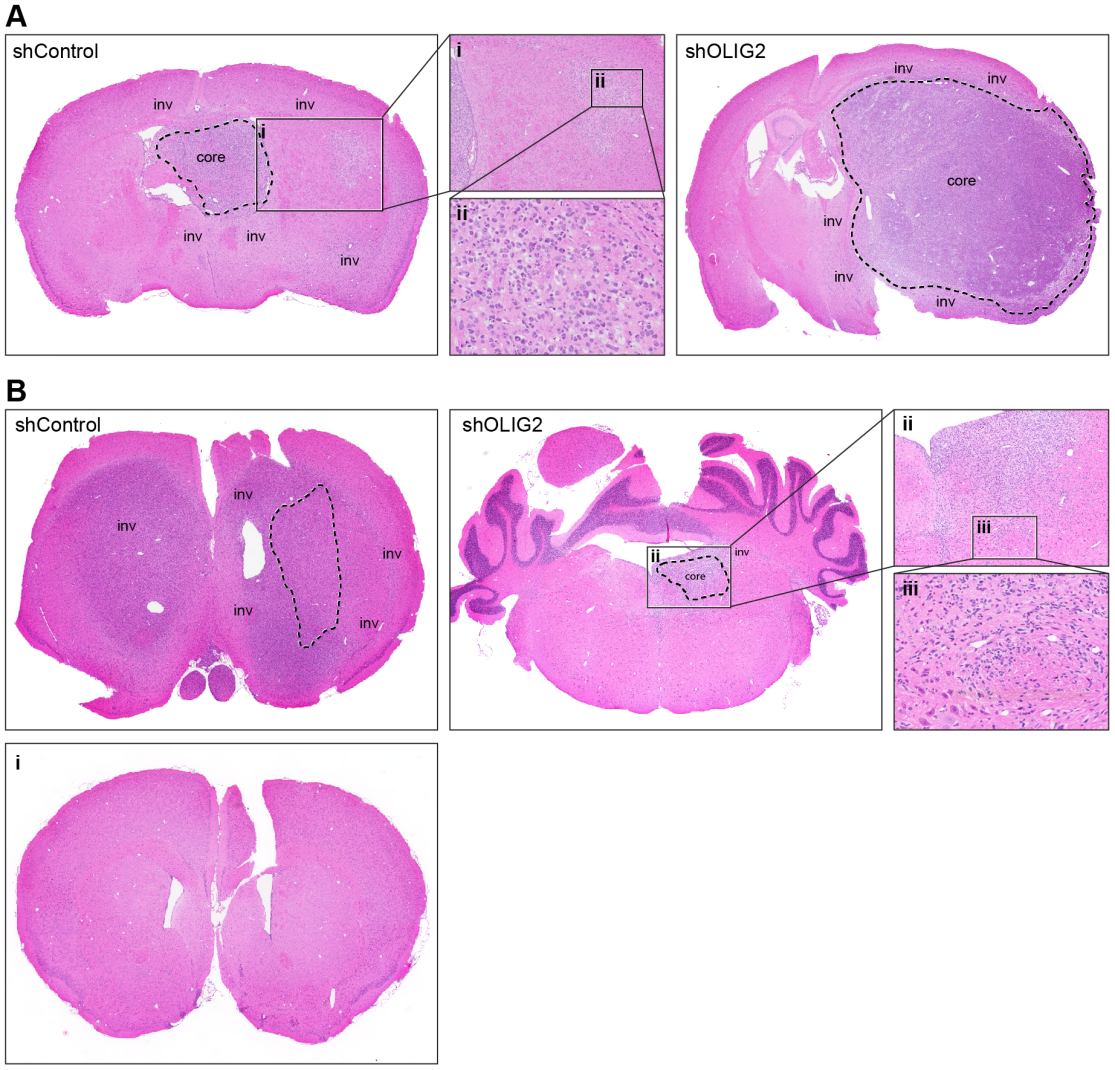
Minimal invasion into adjacent parenchyma in OLIG2 knockdown GSCs. GSCs transduced with a shOLIG2 lentivirus or a control lentivirus were implanted intracranially into SCID mice. OLIG2 knockdown results in reduced tumor invasion in both (A) 0923 and (B) 1228A1 GSCs (n = 6 animals per group), intracranial tumor histology (H&E, scale bar, 100 µm). shControl and shOLIG2 as designated, dashed line indicates the tumor core. (Ai) Solid line box at the AOI of 0923 shControl GSCs, Magnification ×4. (Aii) Solid line box, Magnification ×20. (Bi) Anterior section of 1228A1 of shOLIG2 (no indication of tumor cells). (Bii) Solid line box at the AOI of 1228A1 shOLIG2 GSCs, Magnification ×4. (Biii) Solid line box, Magnification ×20.

Finally, we evaluated the effect of shOLIG2 on glioma invasion *in vivo*. Figure 7 demonstrates that while the total number of inoculated GSCs is similar in shControl and shOLIG2 xenograft tumors, the shOLIG2 is confined to a solitary mass with minimal invasion into adjacent parenchyma. Interestingly, both 0923 and 1228A1 shControl xenografts (Figures 7A and 7B, accordingly) exhibited significantly more widespread invasion, including to the contralateral hemisphere.

## Discussion

Surgical resection of gliomas is usually non-curative secondary to the invasive nature of the tumor [4] and thus therapeutic targeting of IGCs is an attractive concept. This study presents a unique approach that led to the simultaneous identification of genes in glioma cells and brain microenvironment at the AOI *in situ*, which enabled us to construct and evaluate pathways that may underlie glioma invasion as both a cell autonomous and tumor-host interactive process.

Previous studies of global gene expression in GBM invasion used LCM of patient specimens did not distinguish changes in the microenvironment at the site of invasion [8], [9]. Although the cell type-specific gene expression analysis of complex tissues was previously proposed, it is not applicable with complex solid tissues such as brain bearing tumor [36]. Our novel approach of LMD enrichment and microarray analysis in GSCs xenografts allowed us to simultaneously explore the DEGs of both IGCs and host microenvironment. For instance, while Hoelzinger et al. [8] reported an increased expression of *EFNB3* in the tumor rim, we found it to be specifically upregulated in the microenvironment rather than in the invasive tumor cells, a distinction key to understanding complex cellular invasion pathways. In order to measure tumor and microenvironment specific gene expression at the AOI, we distinguished human and mouse specific microarray signals within the mixed sample by eliminating individual probes susceptible to cross-hybridization. The specificity of the results was validated based on the number of DEGs using a species-specific assay (NanoString). These probes were designed to only capture human or mouse genes, and a significant (70–80%) proportion was indeed validated, and is in line with previous reports using pure samples [37].

Many of the DEGs common for both xenografted GSCs were at a low fold-change, but were nonetheless statistically significant. We then looked at the intersection of our DEGs with those previously reported in the invasive rim of 4 human GBMs in an attempt to correlate our xenograft data to a closely related one derived in a clinical setting [9]. Although not entirely comparable as our invasive cells are collected at some distance from the tumor rim, this approach resulted in an overlap of 127 genes (Table S6 in File S1). Genes such as *CDK1*, *BUB1*, *FBX05*, *ADAM23* and *PLXNA4*, associated with cell cycle, cell adhesion or neurophysiological processes were found in both datasets. Though upregulated genes within the cell cycle functional category can imply activation of cellular proliferation, we observed the presence of inhibitory proteins of APC such as *BUB1* and *FBX05*, which could promote cell cycle arrest [15], [17]. The downregulation of *PLXNA4*, a semaphorin receptor required for guidance of newly differentiated neurons, was also common to both datasets [18]. Gene ontology and pathway mapping for gene function have a crucial role in translating gene expression data into models of biological function [38]. Interestingly, our glioma dataset highlights the down-modulation of EMT related genes that implicate a phenotypic shift toward a mesenchymal-epithelial transition (MET) as a major process undergone by tumor cells at the AOI. Previous genomic profiling of metastatic tumors has shown that the disseminated tumor cells are strikingly similar to primary tumor cells [39], [40]. These studies suggested that phenotypic transitions might be driven not only by cell intrinsic affecters but also by the influence of the microenvironment. Indeed, the present study describes a tumor invasion-specific microenvironment functional network that further supports this idea.

Being exploratory and hypothesis generating, our analysis suggests some potential protein-protein interactions that have never been explored between glioma cells and host cells at the AOI. Recently the role of *EPHA2* and *EPHA3* in maintaining the undifferentiated state of GSC has been reported [41], [42]. Our data shows *EFNB3* enrichment at the AOI accompanied by *EPHA4* expression in the IGCs. Cell contact-mediated bidirectional *EFNB3/EPHA4* signaling and migration of interneurons during cerebral cortex development has been previously reported, suggesting the possible usage of this interaction during glioma invasion [20]. Expression of *EFNB3* in the cerebral cortex may provide the switch between cell-cell adhesion and cell migration by A-Disintegrin-And-Metalloproteases (ADAM)-mediated shedding [43]. Intriguingly, we observe that IGCs, host cells or both, show increased expression of *ADAM10*, *ADAM15* and *ADAM19* at the AOI.

The presence of *OLIG2* positive tumor cells within the AOI strongly suggests that the invasive niche contains GSCs [35]. Knockdown of *OLIG2* in 0923 GSCs decreased the expression levels of their NSC markers (*SOX2*, *NANOG*, *OCT4* and *NESTIN*), and reduced their self-renewal and proliferation abilities. Exposure of these cells to differentiation conditions reveals the potential of the shOLIG2 GSCs to differentiate more profoundly into distinct populations of glial and neuronal lineages than GSCs which express *OLIG2*. Moreover, *OLIG2* knockdown inhibited their migration potential *in vitro* and *in vivo*. Recently, Siebzehnrubl et al. [44] demonstrated that critical stem cell regulators such as *SOX2* and *OLIG2* are induced by *ZEB1-miR-200* feedback loop in GBM. Our data support their model, as non-invasive cells in the tumor mass express relatively low levels of *OLIG2*. Our *in vitro* data further show that *OLIG2* can modulate *TWIST* and *SNAI2* protein expression, the central regulators of EMT cancer invasion [45]. Consistently, our microarray data also show a close correlation between *OLIG2*, *TWIST* and *SNAI2* expression within the AOI of xenografted 0923 GSCs.

In epithelial cancer, EMT is evoked during tumor cell invasion and metastasis, leading to the generation of cancer cells with stem cell-like characteristics. Metastases are accompanied by a re-differentiation and a MET type transition, both of which are proposed to be a driving force of metastasis, suggesting that MET allows for growth and colonization of the invasive cell [46]. Phillips et al. previously suggested that shifts towards the mesenchymal phenotype in glioma tumors are a pattern of disease progression similar to EMT in epithelial tumor types. We found markers of the mesenchymal phenotype to be downregulated in the IGCs including *CHI3L1* (*YKL40*), *CD44*, and *STAT3* [2]. As EMT is a transient state, once a cell has invaded, its mesenchymal features disappear. Thus, it is likely that the AOI *in situ* contains both invasive and post-invasive (colonizing) glioma cell populations. We postulate that as cells exit the tumor mass and infiltrate brain parenchyma, *OLIG2* expression is enhanced, and as they colonize brain parenchyma, expression of EMT markers is diminished.

While our approach of filtering out cross-hybridizing genes may have resulted in the loss of some differentially expressed genes, the exploratory nature of this study enabled us to capture for the first time multiple pathways that take place at the junction of tumor invasion and microenvironment *in situ*. This study revealed several distinct glioma pathways as well as many previously characterized developmental and neurophysiological processes that had never been described in glioma. These multiple pathways serve as a rich reservoir for therapeutic targets that warrant further investigation. Firstly, our data shows that direct targeting of proteins such as *CD44* [47], *CHI3L1* [48] or *PARD3* may be ineffective at this stage of disease as they are already downregulated in glioma cells at the AOI. On the other hand, the identification of molecules selectively expressed by invasive glioma cells such as *OLIG2* may allow the development of therapeutic strategies that specifically target this population of cells [49]. Furthermore, molecules selectively expressed by host cells within the AOI such as *EFNB3* may be equally amenable to therapeutic targeting.

Specific therapeutic targeting of glioma invasion is a field in its infancy. Within the tumor-bearing CNS, complex, dynamic and synchronized interactions between glioma tumor cells and tumor-microenvironment residing cells (glial, neural and endothelial cells) may sustain glioma cell invasion. Recently, Sottoriva et al. detected the multiple coexisting cell lineages and expression subtypes at the individual GBM patient. They also suggested that these IGCs are a heterogeneous population of malignant cells that survived treatment [50]. Further studies are required in order to determine if invasion is mediated by clonal selection and/or global expression changes. The substantial degree of confirmation at both transcript and protein levels in xenografted GSCs and patient samples, as well as the functional proof of concept, strengthen our phenotypic observations. The data summarized here emphasize the importance of deciphering and understanding the global phenotypic and functional complexity of glioma cell invasion and its dynamic nature for the development of effective glioma treatments in the future.

## Supporting Information

Figure S1 **Migration** ***in vitro*** **and** ***in vivo*** **of 0827A2 GSCs.** (A) Migration activity of 0827A2 GSCs was tested using the xCELLigence RTCA system. The bars represent the average cell indices at 13 h for the indicated conditions of at least 3 experiments. Asterisks indicate significant (*p≤0.05, **p≤0.01) differences in the migration of the cells compared to the control [cells in NBE medium in both upper and lower chambers (NBE/NBE) in uncoated well (−/−)] as determined by *t*-test. Error bars indicate standard error of the means. (B) Migratory nature of xenografted 0827A2 GSCs. H&E stained section depicting restricted infiltration of GBM cells. Inset depicts 10× magnification, representative infiltration area. (C, D) Unsupervised hierarchal clustering in (C) human and (D) mouse arrays of 0827A2 GSCs xenografts shows that the regions are not completely segregated while a clean separation was observed in human and mouse arrays of both 0923 and 1228A1 GSCs xenografts (demonstrated in Figure 1F and 1G, respectively).
(TIF)

Figure S2 **Gene ontology and pathway analysis of host cells at the area of invasion (tumor microenvironment).** (A) Gene ontology analysis of the microenvironment dataset. Please see legend of Figure 2. (B) The tumor microenvironment network at the area of invasion was generated using IPA. Red and green nodes indicate up- and down-regulated genes, respectively. (C) Differentially expressed genes in the tumor microenvironment validated by NanoString.
(TIF)

Figure S3 **Distinguishing invading human GSCs from mouse cells.** Frozen section of xenograft glioma derived from intracranial injection of 1228A1 GSCs were stained with OLIG2 (red), hNuclei (green) and DAPI (blue). Invading GSCs were distinguished by either nuclear size or human-nuclear staining (white arrows). Magnification ×40.
(TIF)

Figure S4 ***OLIG2*** **,** ***PARD3*** **and** ***Efnb3*** **expression in xenografted 1228A1 GSCs.** Frozen sections of xenograft glioma derived from intracranial injection of 1228A1 GSC were stained with (A) *OLIG2*, (B) *PARD3* or (C) *Efnb3* (all in red). DNA was stained with DAPI (blue). At the left side of each panel: intracranial tumor histology (H&E, scale bar, 100 µm) and when available a whole brain tile at same scale. Solid line box (tumor core) and dashed line box (area of invasion) identify magnified (×40) images on right as indicated.
(TIF)

Figure S5 ***EPHA4*** **,** ***WISP1*** **and** ***Shh*** **expression in xenografted GSCs.** Frozen sections of xenograft gliomas derived from intracranial injection of 0923 (Upper panel) or 1228A1 (Lower panel) GSCs were stained with (A) *EPHA4*, (B) *WISP1* or (C) *Shh* (all in red). Please see legend of Figure S4.
(TIF)

Figure S6 **Downregulated expression of EMT associated genes in invasive glioma cells of xenografted 1228A1 GSCs.** Frozen sections of xenograft glioma derived from intracranial injection of 0923 GSC were stained with (A) *CD44*, (B) *CHI3L1* or (C) *ITGA6* (all in red). Please see legend of Figure S4.
(TIF)

Figure S7 ***PDGFA*** **and m** ***Pdgfra*** **expression in xenografted GSC.** Frozen sections of xenograft glioma derived from intracranial injection of 0923 (Upper panel) or 1228A1 (Lower panel) GSC were stained with (A) *PDGFA* and (B) m*Pdgfra* (both in red). Please see legend of Figure S4. Solid line (tumor core), dashed line (invasive area) and dotted line (“normal” cortex area) boxes identify magnified (×40) images on right indicated.
(TIF)

Table S1 **NanoString Codeset Details.**
(XLS)

File S1 Table S2: Pathway Maps in Invading GSCs and the Tumor Microenvironment. Table S3: Potential Protein-Protein Interactions between Invading GSCs and Their Microenvironment Residing Cells. Table S4: Differentially expressed genes in invading GSCs (array data). Table S5: Differentially expressed genes in the tumor microenvironment (array data). Table S6: Glioma invasion-related genes common with previously reported study by Kislin et al. (reference [9] in the paper).
(DOC)
