# Supplementary figures and images for: Identification of Molecular Pathways Facilitating Glioma Cell Invasion In Situ
Source: PLoS One. 2014 Nov 3;9(11):e111783. doi: 10.1371/journal.pone.0111783 (PMC4218815; doi:10.1371/journal.pone.0111783)

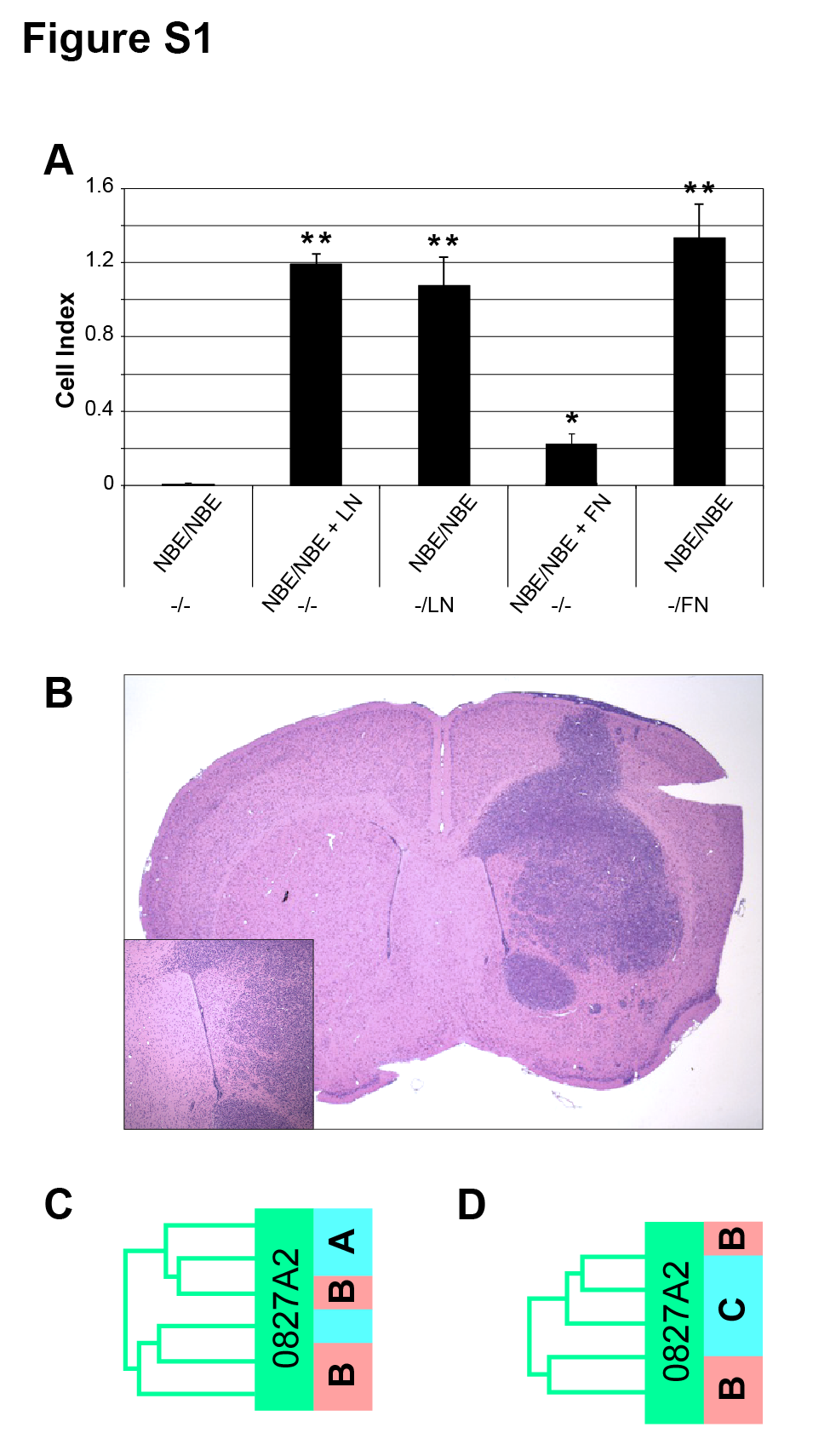

Supplement: Figure S1 — Migration in vitro and in vivo of 0827A2 GSCs. (A) Migration activity of 0827A2 GSCs was tested using the xCELLigence RTCA system. The bars represent the average cell indices at 13 h for the indicated conditions of at least 3 experiments. Asterisks indicate significant (*p≤0.05, **p≤0.01) differences in the migration of the cells compared to the control [cells in NBE medium in both upper and lower chambers (NBE/NBE) in uncoated well (−/−)] as determined by t-test. Error bars indicate standard error of the means. (B) Migratory nature of xenografted 0827A2 GSCs. H&E stained section depicting restricted infiltration of GBM cells. Inset depicts 10× magnification, representative infiltration area. (C, D) Unsupervised hierarchal clustering in (C) human and (D) mouse arrays of 0827A2 GSCs xenografts shows that the regions are not completely segregated while a clean separation was observed in human and mouse arrays of both 0923 and 1228A1 GSCs xenografts (demonstrated in Figure 1F and 1G, respectively). (TIF) [file pone.0111783.s001.tif]

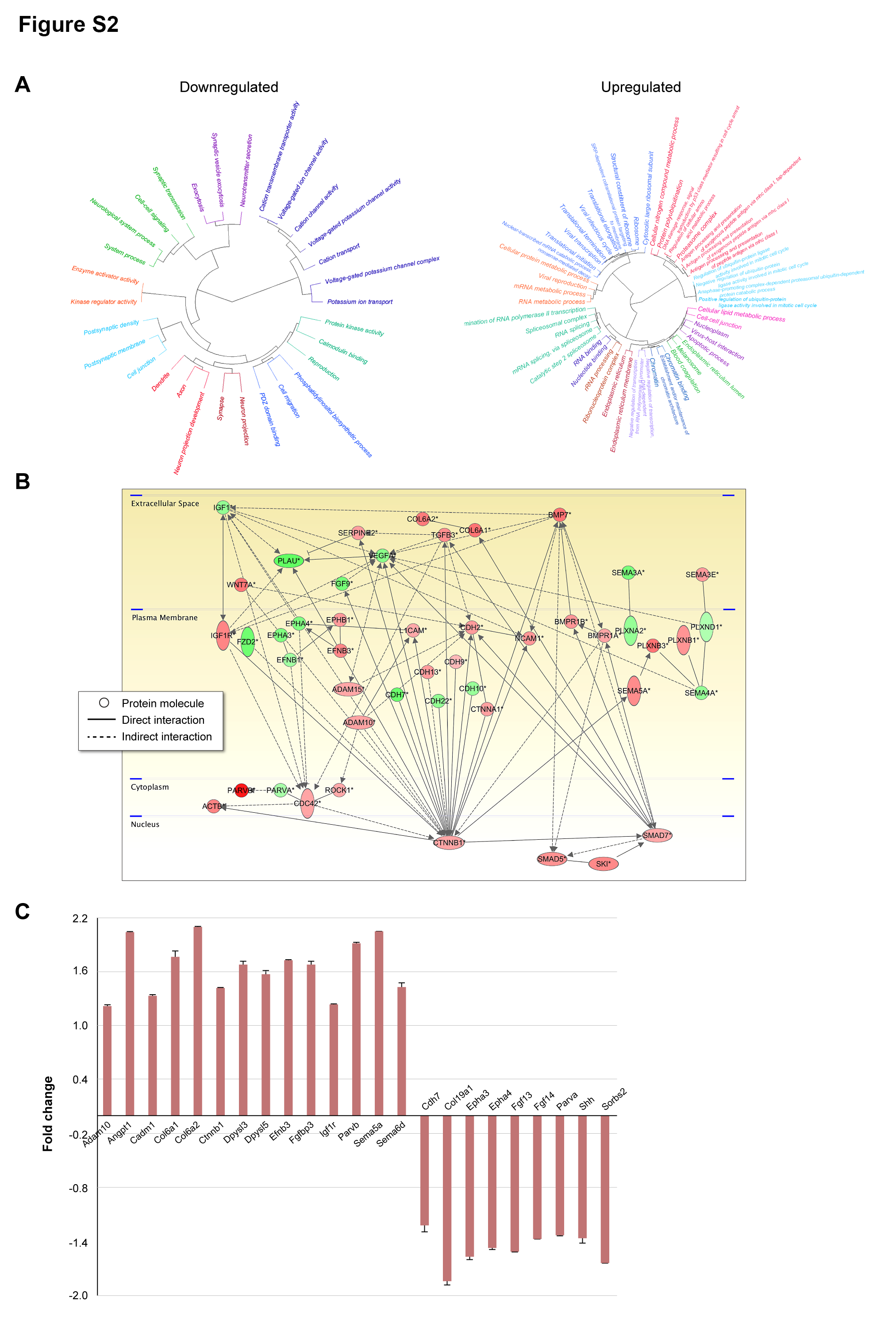

Supplement: Figure S2 — Gene ontology and pathway analysis of host cells at the area of invasion (tumor microenvironment). (A) Gene ontology analysis of the microenvironment dataset. Please see legend of Figure 2. (B) The tumor microenvironment network at the area of invasion was generated using IPA. Red and green nodes indicate up- and down-regulated genes, respectively. (C) Differentially expressed genes in the tumor microenvironment validated by NanoString. (TIF) [file pone.0111783.s002.tif]

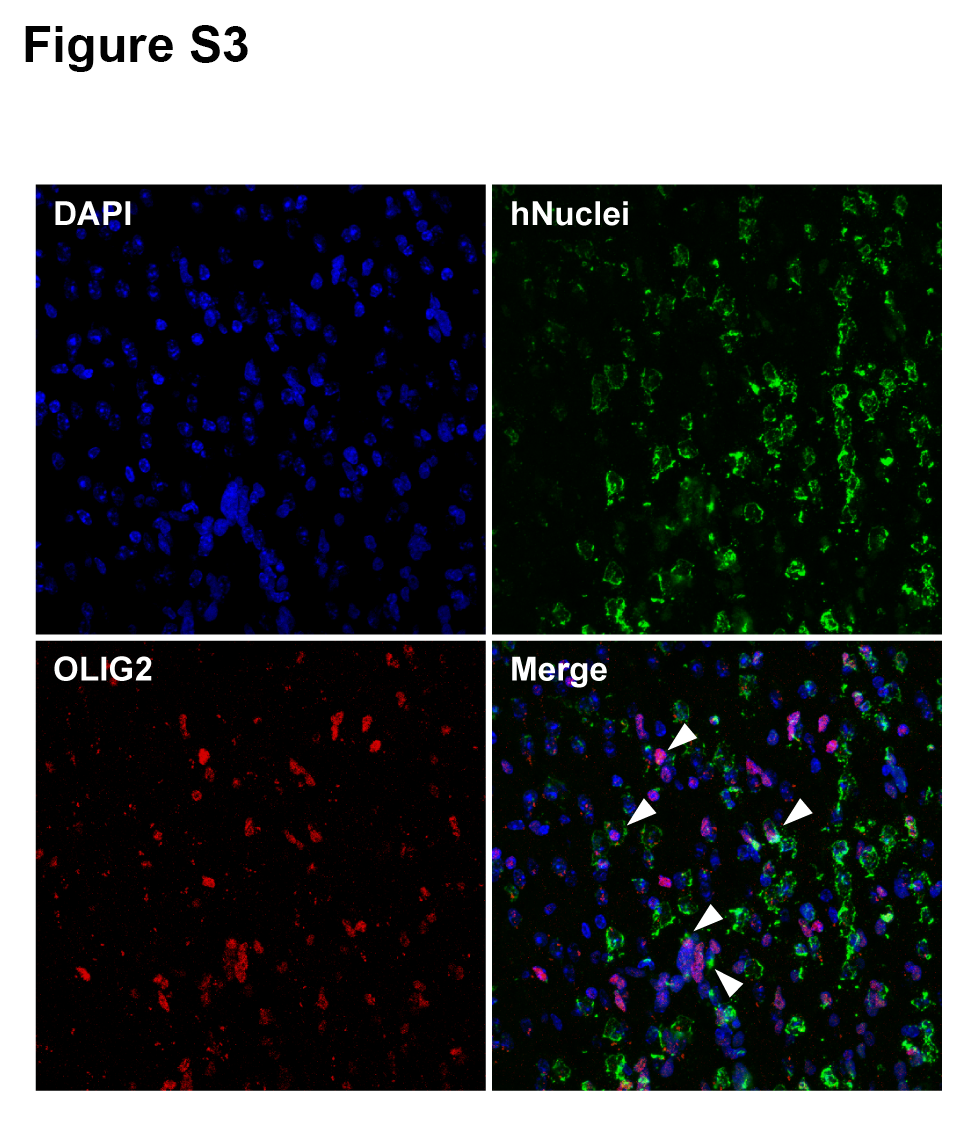

Supplement: Figure S3 — Distinguishing invading human GSCs from mouse cells. Frozen section of xenograft glioma derived from intracranial injection of 1228A1 GSCs were stained with OLIG2 (red), hNuclei (green) and DAPI (blue). Invading GSCs were distinguished by either nuclear size or human-nuclear staining (white arrows). Magnification ×40. (TIF) [file pone.0111783.s003.tif]

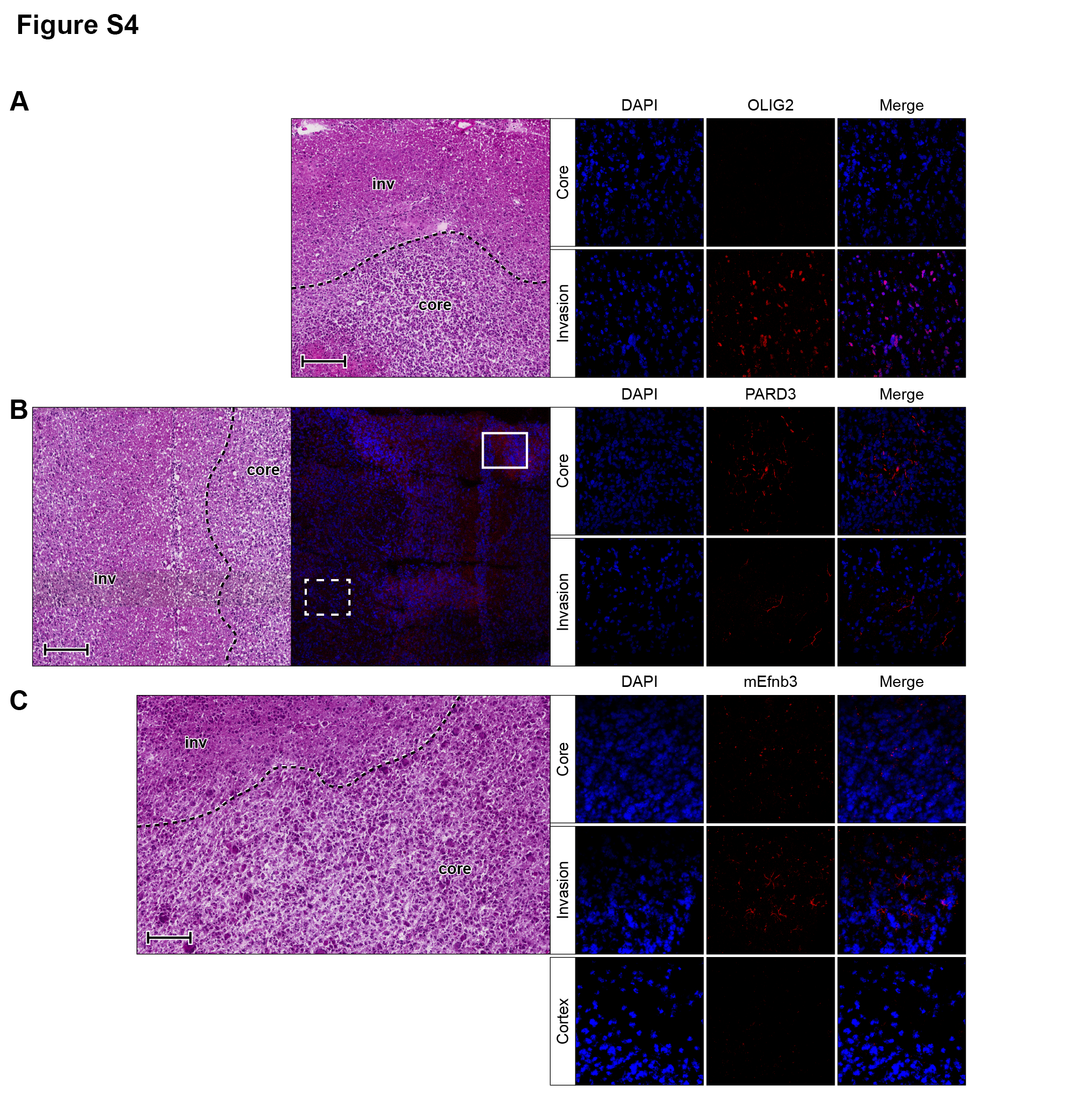

Supplement: Figure S4 — OLIG2 , PARD3 and Efnb3 expression in xenografted 1228A1 GSCs. Frozen sections of xenograft glioma derived from intracranial injection of 1228A1 GSC were stained with (A) OLIG2, (B) PARD3 or (C) Efnb3 (all in red). DNA was stained with DAPI (blue). At the left side of each panel: intracranial tumor histology (H&E, scale bar, 100 µm) and when available a whole brain tile at same scale. Solid line box (tumor core) and dashed line box (area of invasion) identify magnified (×40) images on right as indicated. (TIF) [file pone.0111783.s004.tif]

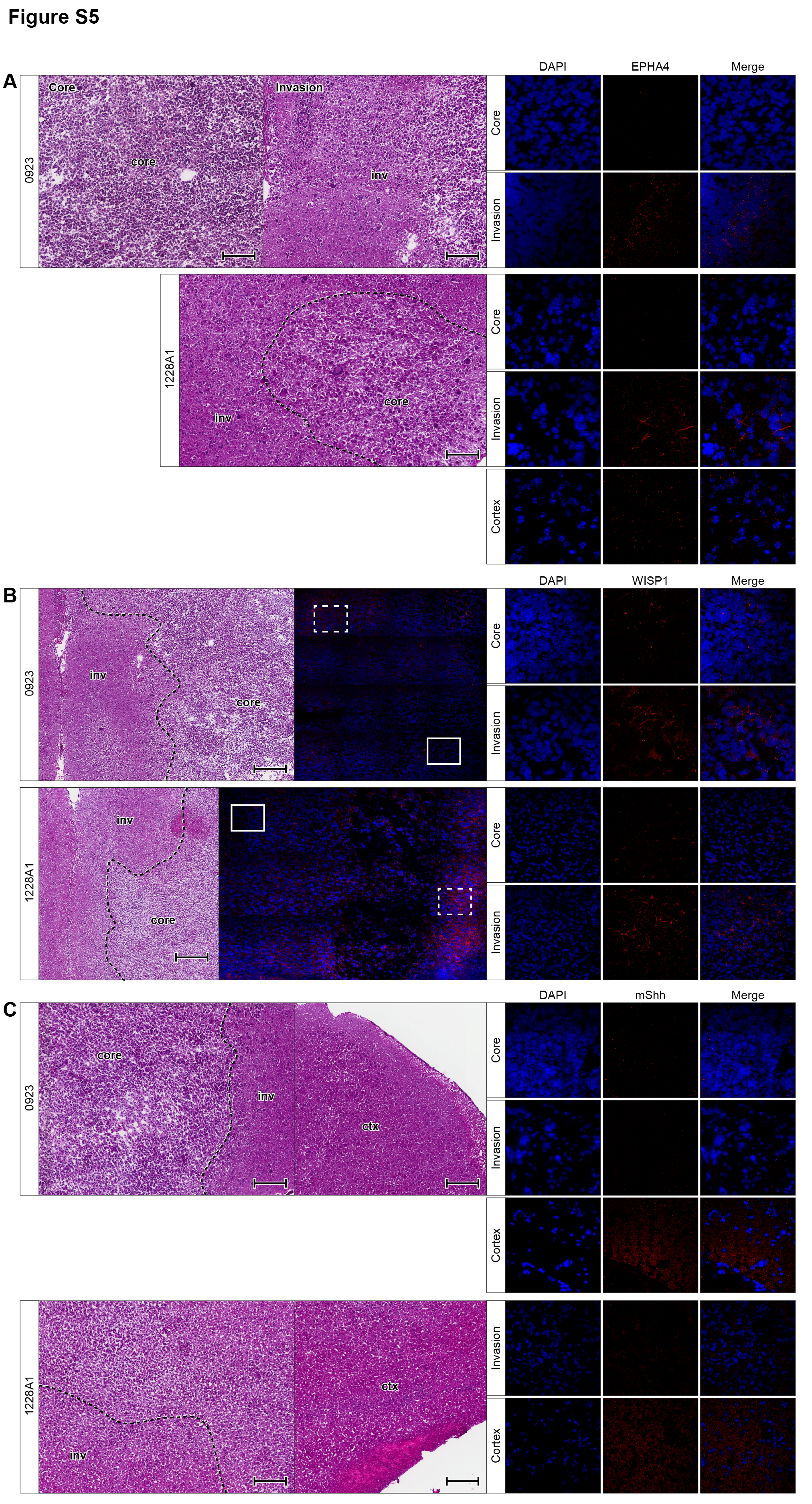

Supplement: Figure S5 — EPHA4 , WISP1 and Shh expression in xenografted GSCs. Frozen sections of xenograft gliomas derived from intracranial injection of 0923 (Upper panel) or 1228A1 (Lower panel) GSCs were stained with (A) EPHA4, (B) WISP1 or (C) Shh (all in red). Please see legend of Figure S4. (TIF) [file pone.0111783.s005.tif]

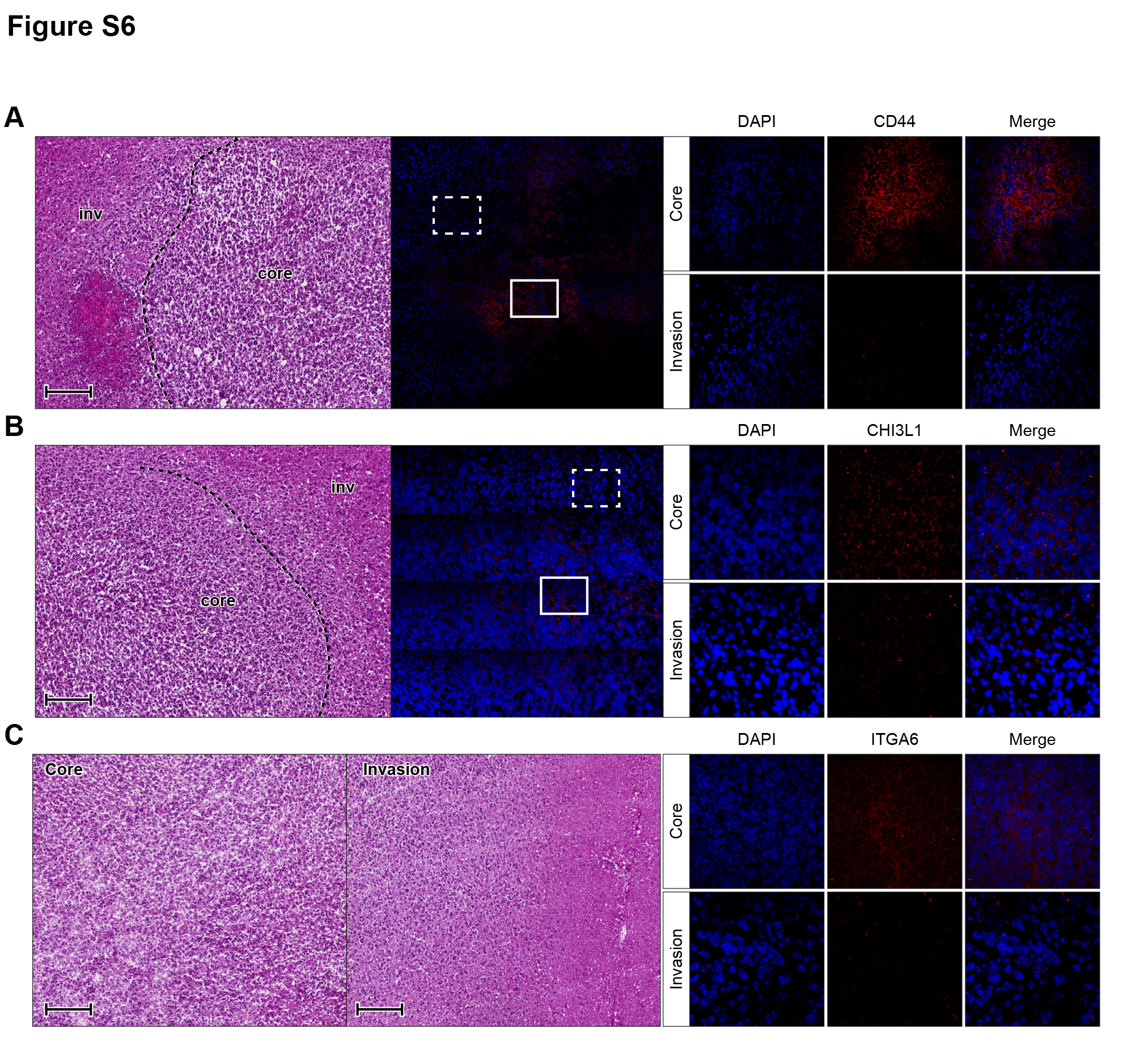

Supplement: Figure S6 — Downregulated expression of EMT associated genes in invasive glioma cells of xenografted 1228A1 GSCs. Frozen sections of xenograft glioma derived from intracranial injection of 0923 GSC were stained with (A) CD44, (B) CHI3L1 or (C) ITGA6 (all in red). Please see legend of Figure S4. (TIF) [file pone.0111783.s006.tif]

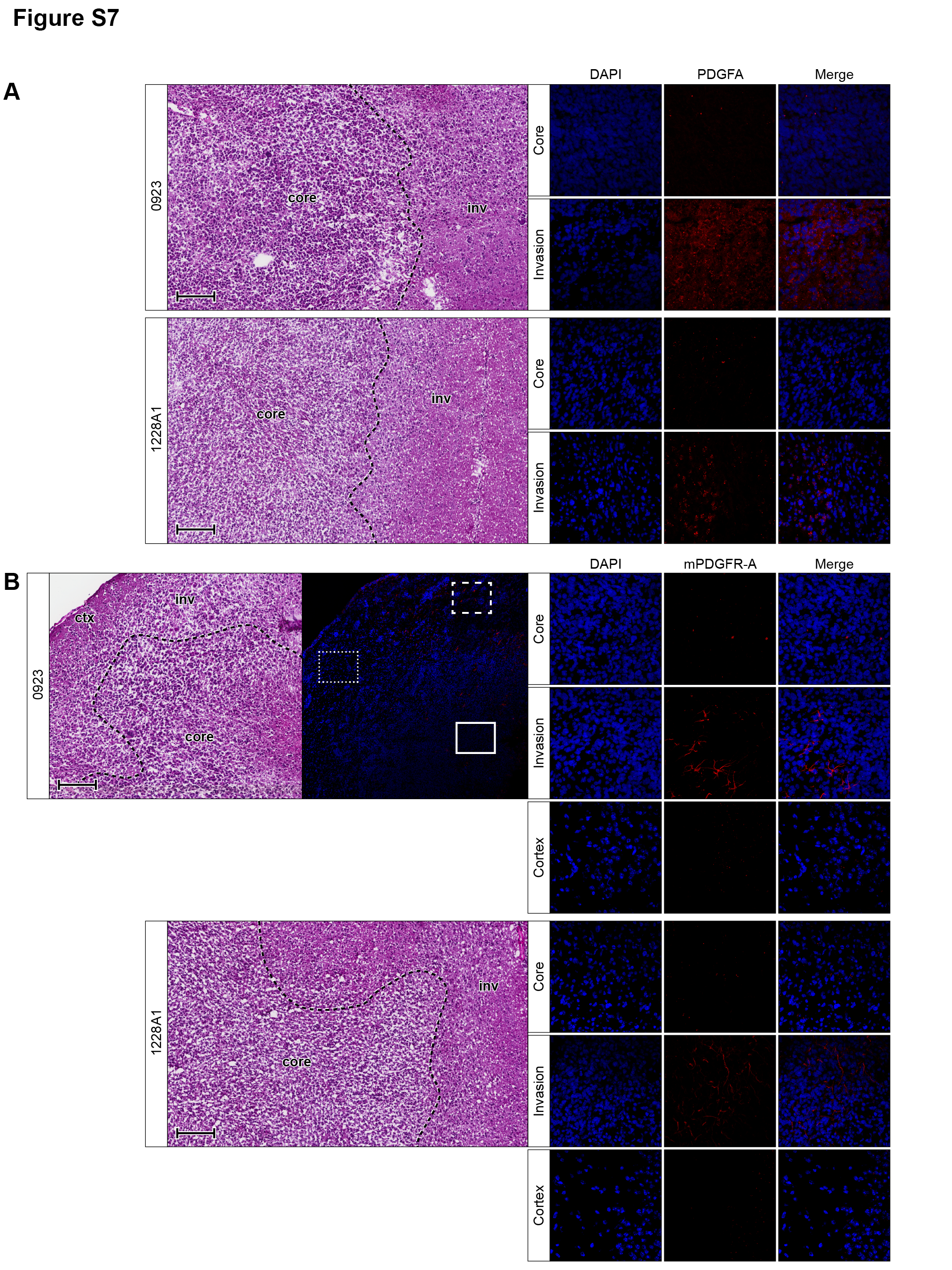

Supplement: Figure S7 — PDGFA and m Pdgfra expression in xenografted GSC. Frozen sections of xenograft glioma derived from intracranial injection of 0923 (Upper panel) or 1228A1 (Lower panel) GSC were stained with (A) PDGFA and (B) mPdgfra (both in red). Please see legend of Figure S4. Solid line (tumor core), dashed line (invasive area) and dotted line (“normal” cortex area) boxes identify magnified (×40) images on right indicated. (TIF) [file pone.0111783.s007.tif]
