# Supplementary material for: Identification of Molecular Pathways Facilitating Glioma Cell Invasion In Situ
Source: PLoS One. 2014 Nov 3;9(11):e111783. doi: 10.1371/journal.pone.0111783 (PMC4218815; doi:10.1371/journal.pone.0111783)
Supplement: File S1 — Table S2: Pathway Maps in Invading GSCs and the Tumor Microenvironment. Table S3: Potential Protein-Protein Interactions between Invading GSCs and Their Microenvironment Residing Cells. Table S4: Differentially expressed genes in invading GSCs (array data). Table S5: Differentially expressed genes in the tumor microenvironment (array data). Table S6: Glioma invasion-related genes common with previously reported study by Kislin et al. (reference [9] in the paper). (DOC) [file pone.0111783.s009.doc]

**Supporting Information**

#### Materials and Methods

**Cell Lines**

Human glioblastoma U87MG cells and HEK293T cells were obtained from the American Type Culture Collection (ATCC; Manassas,VA) and were cultured in Dulbecco’s modified Eagle medium (DMEM; Gibco, Carlsbad, CA) containing 5-10% fetal bovine serum (FBS), 1% L-glutathionine, and 1% penicillin–streptomycin (all from Gibco). RNA from the U87MG cell line was used as a microarray batch effect control. Medium containing serum was used for differentiation assays.

**Real-Time Measurement of Cell Migration**

GSCs migration was assessed by the xCELLigence RTCA DP device. Briefly, 100,000 cells were seeded per well in CIM-Plates 16 which were precoated (haptotaxis) on the underside membrane of the upper chamber with 10ug/ml Laminin or 20ug/ml Fibronectin (Sigma, St. Louis, MO). For some wells, these factors were added to the culture medium at the lower chamber without coating the underside membrane (chemotaxis). NBE medium was added to both lower and upper wells. Data acquisition and analysis was performed with the RTCA software (version 1.2, Roche Diagnostics).

**Human Tissues**

Following signed informed consent, tumor tissue was obtained from patients (aged ≥18 years) undergoing medically indicated resection of malignant gliomas at the National Institutes of Health as part of a clinical trial approved by the Institutional Review Board (NCI-02C0140). The most recent MRI was used by the pathologist to guide the autopsy procedure. Tumor core as well as brain regions that were macroscopically infiltrated by the tumor were identified, registered, photographed and fixed in paraformaldehyde (PFA) to determine the histological diagnosis. Each patient presented a different spatial distribution of invasive cells relative to the tumor core. The invasive areas sampled represent regions separated from the visible tumor bulk, containing no gross visible disease, but containing microscopic neoplastic cells.

**Lentiviral Vector Preparation**Lentiviruses were produced in 293T following transfection using Lipfectamin 2000 (**Life Technologies**) with OLIG2 shRNA Plasmid or Control shRNA plasmid (Santa Cruz Biothecnology Inc.) and packaging plasmids psPAX2 and pMD2.G (Addgen, MA) according to the manufacturer's instructions. **Limiting Dilution and Proliferation Assay**
For the limiting dilution assay, cells were plated into 96-well plates at 2 and 5 cells per well and were incubated at 37°C for 10 days. Each well was examined for the formation of spheres. For the proliferation assay, cells were plated into 6-well plates with 1 X 10^5^ cells per well and incubated at 37°C for 2, 4 and 6 days. At the time of quantification, cells in each well were counted using Vi-CELL XR (Beckman Coulter, Inc., CA).

**Laser Micro-Dissection (LMD) and RNA Isolation**Brains were handled in RNase free conditions, imbedded in OCT compound (Sakura Finetek, CA), frozen immediately and kept at -80°C. Cryopreserved glioma xenografts were sectioned at the point of full cut section, in a serial 10μm sections and mounted on MembraneSlides PEN-membrane 2μm (Leica Microsystems CMS GmbH, Germany) in RNase free conditions. All slides were stored at -80°C until LMD was completed. For each evaluated stains, the same staining procedures were performed. Fixation was directly performed avoiding sections to defrost by incubating slides 1 min into 95% ethanol. Staining solution was then directly applied to the section and incubated for 1min, followed by rinsing in 95% ethanol in two steps (1 min each), dehydration in 100% ethanol in two steps (2 min each), and xylene in two steps (9 min each). Fresh solutions were used for each staining. Staining solution was prepared as previously described.
Slides were air dried under laminar flow and immediately processed for LMD using the Leica LMD6000 (Leica Microsystems CMS GmbH). Specimens were collected by gravity, contact-free and contamination-free into tubes containing lysis buffer. Three distinct regions were collected: (1) regions enriched with tumor cell infiltration, containing 75-80% infiltrative tumor cells, (white matter) with reactive cells within the infiltrative population, (2) tumor core area, and (3) regions of reactive mouse cells far from the tumor (collateral hemisphere). Total cellular RNA isolation from LMD brain samples as well as from GSC lines was carried out using RNAqueous®-Micro (Ambion, TX) and further purified using RNeasy MinElute Cleanup Kit (QIAGEN Inc., CA).

**Gene Expression Microarray Analysis**
The quality control of RNA preparations and the assessment of RNA amount were carried out using NanoDrop 8000 (Thermo Scientific, DE) and Agilent 2100 Bioanalyzer (Agilent, Santa Clara, CA). For all the samples the RNA Integrity Number for the total RNA was greater than 7. The total mRNA was amplified using GeneChip® 3’ IVT Express Kit (Affymetrix, Santa Clara, CA). Briefly, 100 ng of total RNA was initially converted into first-strand cDNA and then double-stranded DNA template was generated. Biotin-Conjugated amplified RNA (aRNA) was synthesized by *in vitro* transcription from the DNA template. The purified biotin-labeled aRNA was fragmented and hybridized onto Affymetrix HG-U133 plus2 or Mouse430_2 GeneChip Arrays, according to the Affymetrix GeneChip Expression Analysis Technical Manual (Affymetrix). All arrays were confirmed to be within acceptable minimal quality control parameters including the signal intensity ratio of the 5′ and 3′ ends of the internal control genes of β-actin and GAPDH less than 2 with exception for arrays designed for cross-species hybridization. The initial gene expression analysis data files (CEL files) were generated using Affymetrix Expression Console Software version 1.1.

**Generation of alternative chip definition files (cdf’s) for mixed species sample analysis**To find genes differentially expressed at the area of invasion relative to the tumor core, RNA samples taken from the invasive area from four mice were compared to the paired tumor cores within each animal and hybridized on human arrays. Correspondingly, for the analysis of genes differentially regulated in the microenvironment, RNA at the invasive areas and distal mouse brain from the same animal was hybridized on mouse microarrays. In order to reduce the influence of cross-reactive signal of mouse RNA on human gene expression data taken from mixed samples at the area of invasion, individual microarray probes were identified as having high or low cross-reactivity against mouse RNA by hybridizing human microarrays with RNA extracted from mouse brain. Using raw intensity signals from cel files, individual probes were filtered if they met the following criteria: the net intensity (PM-MM) of each probe pair from the cross-reactive species was less than 10 and greater than 50% of the corresponding signal from human tumor core. Alternatively, to detect cross-reactive human signal on the mouse microarrays, mouse chips were hybridized with pure human cell line RNA and the corresponding same criteria were applied. The remaining probes were grouped into their original probe sets and those that had at least 4 remaining probes were retained in the new chip definition file (cdf). This resulted in alternative cdf’s containing 26,954 probe sets for the HG-U133 plus2 array and 21,675 probe sets for the Mouse430_2 array.


**Differential gene expression analysis and pathway analysis**The gene expression data were calculated using the MAS5 algorithm following quantile normalization, and the results subjected to statistical analysis using a two-way Anova. In order to limit the number of false positives, we prioritized genes that were commonly changed in both cell lines at p<0.05 for follow up validation, which would markedly reduce the number of genes that are changed by chance alone (false discovery). In addition, some other genes were selected based on biological interest for further validation by the NanoString assay. Global gene ontology enrichment analysis was performed on up- and downregulated genes which had p values < 0.05, using the hypergeometric distribution. The three ontology domains were included in the analysis. Additional pathway analysis was performed on the significant differentially expressed genes using the MetaCore database (Thomson Reuters). Pathway maps comparison analysis between the individual GSC datasets was performed separately for human or mouse DEGs. A p value was calculated for each gene group mapped to certain pathways using the hypergeometric distribution to determine the probability that the association between the genes and each biological pathway was not explained by chance alone. Significant genes that met filter criteria were picked to form a global molecular network. Networks were graphically depicted for visualization of gene relationships using Ingenuity Pathway Analysis (IPA, Ingenuity Systems, Redwood City, CA). Extended analysis using IPA’s Path Explorer tool was performed with DEGs in the plasma membrane and extracellular space in order to highlight potential protein-protein interactions between the invading glioma cells and cells that reside at the microenvironment. Protein-protein interactions that were experimentally validated in at least one publication were listed while cis-interactions were excluded from the list.
 **Histology and Immunofluorescence**Frozen sections (10μm) were prepared and stained with haematoxylin and eosin (Histoserv, Germantown, MD). The immunofluorescence labeling was performed on 30μm frozen sections mounted on charged glass slides or cells plated on Poly-L-ornithine (Sigma) coated slides. Sections were fixed with cold 4% PFA, 100% methanol or methanol:acetone (v/v) for 10 minutes, rapidly rehydrated with phosphate-buffered saline (PBS), blocked and permeabilized with blocking solution (2.5% normal goat serum, 2.5% normal horse serum and 0.25% Triton-x-100) for 1hr at room temperature. Subsequently, sections were incubated overnight at 4°C with the primary antibody. Following washing with PBS, sections were incubated for 1hr at room temperature with the appropriate secondary antibody. For multiple staining of two different mouse primary antibodies on the same tissue section, the M.O.M blocking reagent (MKB-2213, Vector Laboratories, Inc., CA) was used according to the manufacture instructions. The sections were mounted with **VECTASHIELD HardSet Mounting Medium with DAPI** (H-1500, Vector Laboratories, Inc.). Figures 3 and 4 as well as Figures S4-S7 contain a whole brain tile images captured using a mosaic function. Since the DAPI staining do not penetrate equally the condensed tumor and the less condensed tissue we could not always capture all of the single images with good DAPI staining at the same depth level. The H&E staining in the figures shows the presence of a condensed tumor/brain tissue at these areas. A veterinarian pathologist examined histological staining and distinguished invading human glioma cells from other cells by nuclear size or human-nuclei staining (Supplementary Figure S3). Images were captured using a Zeiss LSM 510 confocal microscope.
 **Antibodies**Primary antibodies used for immunofluorescence labeling, immunohistochemistry and Western blotting are: TUJ1 (Covance, Gaithersburg, MD); SOX2 (R&D Systems); GFAP (Dako, Glostrup, Denmark); Nestin (IBL, Gunma, Japan); CHI3L1 (mAY), human-Nuclei (235-1), Olig-2, Sox9 (Millipore, CA); PARD3, SHH (3A2), PDGFA, WISP1, (Novus Biologicals, CO); Integrin alpha 6 (MP 4F10), Ephrin B3 (Abcam Inc., MA); CD44, EphA4 (Thermo Scientific, CA); twist (H-81), SLUG (H-140) (Santa Cruz Biothecnology, TX) and mouse-CD140a (Pdgfra, APA5, BD Biosciences, CA).

**Tables**

**Table S2: Pathway Maps in Invading GSCs and the Tumor Microenvironment**

| **Invading GSCs** | | | | | |
| --- | --- | --- | --- | --- | --- |
| **#** | **Maps** | **pValue (0923, 1228A1)** | **min(pValue)** | **Ratio** | |
| 1 | Cell cycle_The metaphase checkpoint | 4.465e-8, 1.678e-12 | 1.678E-12 | [22](../../../../javascript/ViewExpDataObj.network_options_func(711)%3B) | 36 |
| 2 | Cell cycle_Role of APC in cell cycle regulation | 5.295e-9, 1.944e-12 | 1.944E-12 | [19](../../../../javascript/ViewExpDataObj.network_options_func(472)%3B) | 32 |
| 3 | Cytoskeleton remodeling_Cytoskeleton remodeling | 1.064e-10, 3.978e-4 | 1.064E-10 | [43](../../../../javascript/ViewExpDataObj.network_options_func(714)%3B) | 102 |
| 4 | Cell cycle_Spindle assembly and chromosome separation | 2.674e-5, 7.870e-9 | 7.870E-09 | 15 | 33 |
| 5 | Cytoskeleton remodeling_TGF, WNT and cytoskeletal remodeling | 2.105e-8, 1.203e-5 | 2.105E-08 | [43](../../../../javascript/ViewExpDataObj.network_options_func(715)%3B) | 111 |
| 6 | Cell adhesion_Chemokines and adhesion | 9.562e-8, 9.868e-5 | 9.562E-08 | 39 | 100 |
| 7 | Development_Regulation of epithelial-to-mesenchymal transition (EMT) | 2.059e-7, 6.605e-5 | 2.059E-07 | 26 | 64 |
| 8 | Cell cycle_Chromosome condensation in prometaphase | 4.215e-4, 3.038e-7 | 3.038E-07 | 13 | 21 |
| 9 | G-protein signaling_Regulation of CDC42 activity | 6.357e-7, 1.202e-3 | 6.357E-07 | [16](../../../../javascript/ViewExpDataObj.network_options_func(389)%3B) | 33 |
| 10 | Development_TGF-beta receptor signaling | 1.698e-6, 1.507e-3 | 1.698E-06 | [24](../../../../javascript/ViewExpDataObj.network_options_func(475)%3B) | 50 |
| 11 | Cell cycle_Influence of Ras and Rho proteins on G1/S Transition | 8.035e-5, 6.593e-6 | 6.593E-06 | [25](../../../../javascript/ViewExpDataObj.network_options_func(4583)%3B) | 53 |
| 12 | Development_EGFR signaling pathway | 1.952e-3, 1.236e-5 | 1.236E-05 | [25](../../../../javascript/ViewExpDataObj.network_options_func(443)%3B) | 63 |
| 13 | Cell cycle_Role of Nek in cell cycle regulation | 1.861e-5, 9.662e-4 | 1.861E-05 | [13](../../../../javascript/ViewExpDataObj.network_options_func(731)%3B) | 32 |
| 14 | Cytoskeleton remodeling_Regulation of actin cytoskeleton by Rho GTPases | 2.041e-5, 1.627e-2 | 2.041E-05 | [12](../../../../javascript/ViewExpDataObj.network_options_func(551)%3B) | 23 |
| 15 | Neurophysiological process_Receptor-mediated axon growth repulsion | 2.627e-3, 9.186e-3 | 2.627E-03 | [16](../../../../javascript/ViewExpDataObj.network_options_func(527)%3B) | 45 |
| **Tumor Microenvironment** | | | | | |
| **#** | **Maps** | **pValue (0923, 1228A1)** | **min(pValue)** | **Ratio** | |
| 1 | Cytoskeleton remodeling_TGF, WNT and cytoskeletal remodeling | 3.724e-6, 1.752e-9 | 1.752E-09 | [42](../../../../javascript/ViewExpDataObj.network_options_func(715)%3B) | 111 |
| 2 | Cytoskeleton remodeling_Cytoskeleton remodeling | 1.899e-6, 4.693e-9 | 4.693E-09 | [41](../../../../javascript/ViewExpDataObj.network_options_func(714)%3B) | 102 |
| 3 | Cell adhesion_Ephrin signaling | 3.147e-7, 1.014e-8 | 1.014E-08 | [22](../../../../javascript/ViewExpDataObj.network_options_func(649)%3B) | 45 |
| 4 | Neurophysiological process_NMDA-dependent postsynaptic long-term potentiation in CA1 hippocampal neurons | 2.719e-8, 1.185e-8 | 1.185E-08 | [33](../../../../javascript/ViewExpDataObj.network_options_func(3040)%3B) | 80 |
| 5 | Cytoskeleton remodeling_Reverse signaling by ephrin B | 2.302e-4, 1.346e-8 | 1.346E-08 | [17](../../../../javascript/ViewExpDataObj.network_options_func(529)%3B) | 31 |
| 6 | Cell adhesion_Chemokines and adhesion | 1.288e-5, 2.548e-7 | 2.548E-07 | [33](../../../../javascript/ViewExpDataObj.network_options_func(716)%3B) | 100 |
| 7 | Development_TGF-beta receptor signaling | 1.571e-4, 4.013e-7 | 4.013E-07 | [22](../../../../javascript/ViewExpDataObj.network_options_func(475)%3B) | 50 |
| 8 | Neurophysiological process_EphB receptors in dendritic spine morphogenesis and synaptogenesis | 1.068e-6, 1.813e-4 | 1.068E-06 | [17](../../../../javascript/ViewExpDataObj.network_options_func(528)%3B) | 35 |
| 9 | Neurophysiological process_GABA-A receptor life cycle | 1.109e-6, 1.021e-4 | 1.109E-06 | [16](../../../../javascript/ViewExpDataObj.network_options_func(3075)%3B) | 27 |
| 10 | Development_EGFR signaling pathway | 2.249e-3, 2.562e-6 | 2.562E-06 | [22](../../../../javascript/ViewExpDataObj.network_options_func(443)%3B) | 63 |
| 11 | Development_IGF-1 receptor signaling | 1.668e-5, 4.023e-6 | 4.023E-06 | [21](../../../../javascript/ViewExpDataObj.network_options_func(540)%3B) | 52 |
| 12 | Cytoskeleton remodeling_CDC42 in cellular processes | 4.774e-6, 1.527e-5 | 4.774E-06 | [15](../../../../javascript/ViewExpDataObj.network_options_func(390)%3B) | 22 |
| 13 | Immune response_HMGB1/RAGE signaling pathway | 3.275e-3, 2.558e-5 | 2.558E-05 | [16](../../../../javascript/ViewExpDataObj.network_options_func(6111)%3B) | 53 |
| 14 | Cell adhesion_Cadherin-mediated cell adhesion | 2.064e-4, 7.288e-5 | 7.288E-05 | [14](../../../../javascript/ViewExpDataObj.network_options_func(2122)%3B) | 26 |
| 15 | Development_BMP signaling | 1.788e-3, 1.022e-2 | 1.788E-03 | [11](../../../../javascript/ViewExpDataObj.network_options_func(4519)%3B) | 33 |

**Table S3: Potential Protein-Protein Interactions between Invading GSCs and Their Microenvironment Residing Cells**

| **#** | **Invading GSCs** | **TME Cells** | **References** |
| --- | --- | --- | --- |
| 1 | EGFR | Gpm6b |  |
| 2 | EPHA4 | Efnb3 |  |
| 3 | SEMA5A | Plxnb3 |  |
| 4 | APP | Cntn3 |  |
| 5 | APP | L1cam |  |
| 6 | AQP4 | Dag1 |  |
| 7 | FGFR1 | Ncam1 |  |
| 8 | PLXNA4 | Sema6d |  |
| 9 | BGN | Dag1 |  |
| 10 | FGF2 | Sdc2 |  |
| 11 | FSTL1 | Acvr2b |  |
| 12 | FSTL1 | Bmpr1a |  |
| 13 | KAL1 | Sdc2 |  |
| 14 | APP | Aplp2 |  |
| 15 | CD44 | Lgals8 |  |
| 16 | FZD7 | Wnt7a |  |
| 17 | HLA-B | Aplp2 |  |

| Upregulated | Downregulated |
| --- | --- |

| **Upregulated Genes** | | | **1228** | | **923** | |
| --- | --- | --- | --- | --- | --- | --- |
| **Gene** | **Probe Set** | **RefSeq ID** | **FC** | **pval** | **FC** | **pval** |
| ADAM19 | 209765_at | NM_033274 | 1.26 | 0.141 | 1.56 | 0.043 |
| BUB3 | 201458_s_at | NM_001007793 | 1.19 | 0.052 | 1.17 | 0.025 |
| CCNA2 | 213226_at | NM_001237 | 1.37 | 0.002 | 1.22 | 0.011 |
| CDC20 | 202870_s_at | NM_001255 | 1.41 | 0.028 | 1.61 | 0.004 |
| CDC25A | 204695_at | NM_001789 | 1.31 | 0.093 | 1.46 | 0.035 |
| CDCA3 | 223307_at | NM_031299 | 1.35 | 0.019 | 1.23 | 0.008 |
| CDK1 | 203214_x_at | NM_001170406 | 1.34 | 0.002 | 1.12 | 0.001 |
| EPHA4 | 227449_at | NM_004438 | 1.11 | 0.038 | 1.35 | 0.028 |
| IGFBP3 | 210095_s_at | NM_000598 | 1.41 | 0.035 | 1.98 | 0.023 |
| LEF1 | 221558_s_at | NM_001130713 | 1.17 | 0.051 | 1.64 | 0.014 |
| MELK | 204825_at | NM_014791 | 1.48 | 0.002 | 1.24 | 0.034 |
| OLIG2 | 213825_at | NM_005806 | 1.08 | 0.013 | 1.78 | 0.022 |
| PDGFA | 205463_s_at | NM_002607 | 1.21 | 0.008 | 1.40 | 0.082 |
| PTPRZ1 | 204469_at | NM_002851 | 1.11 | 0.001 | 1.09 | 0.023 |
| PTX3 | 206157_at | NM_002852 | 1.29 | 0.019 | 1.53 | 0.032 |
| RBBP8 | 203344_s_at | NM_002894 | 1.23 | 0.008 | 1.32 | 0.016 |
| ROBO2 | 226766_at | NM_001128929 | 1.07 | 0.760 | 1.44 | 0.023 |
| SOX12 | 204432_at | NM_006943 | 1.25 | 0.028 | 1.43 | 0.032 |
| WISP1 | 206796_at | NM_003882 | 1.75 | 0.004 | 2.37 | 0.108 |
| **Downregulated Genes** | | | **1228** | | **923** | |
| **Gene** | **Probe Set** | **RefSeq ID** | **FC** | **pval** | **FC** | **pval** |
| ACTN1 | 208636_at | NM_001102 | -1.62 | 0.005 | -1.63 | 0.001 |
| CD44 | 204489_s_at | NM_000610 | -4.05 | 0.016 | -2.48 | 0.001 |
| CHI3L1 | 209396_s_at | NM_001276 | -2.81 | 0.001 | -2.26 | 0.003 |
| CTNNA1 | 200764_s_at | NM_001903 | -1.41 | 0.012 | -1.69 | 0.001 |
| EFEMP1 | 201842_s_at | NM_001039348 | -1.84 | 0.022 | -2.79 | 0.030 |
| EZR | 208621_s_at | NM_001111077 | -1.64 | 0.035 | -1.64 | 0.064 |
| HLA-G | 210514_x_at | NM_002127 | -1.21 | 0.031 | -2.38 | 0.025 |
| ITGA6 | 201656_at | NM_000210 | -1.48 | 0.008 | -1.33 | 0.030 |
| PARD3 | 221527_s_at | NM_001184785 | -1.29 | 0.031 | -1.29 | 0.003 |
| PFN2 | 204992_s_at | NM_002628 | -1.32 | 0.002 | -1.45 | 0.001 |
| PLXNA1 | 221538_s_at | NM_032242 | -1.08 | 0.065 | -1.05 | 0.469 |
| PLXNA4 | 1562240_at | NM_001105543 | -1.88 | 0.005 | -3.51 | 0.010 |
| RARRES3 | 204070_at | NM_004585 | -1.62 | 0.016 | -3.37 | 0.025 |
| S100A11 | 200660_at | NM_005620 | -3.94 | 0.024 | -2.48 | 0.001 |
| STAT3 | 225289_at | NM_003150 | -1.37 | 0.034 | -1.35 | 0.010 |

**Table S4: Differentially expressed genes in invading GSCs (array data)**

Table S5: Differentially expressed genes in the tumor microenvironment (array data)

| **Upregulated Genes** | | | **1228** | | **923** | |
| --- | --- | --- | --- | --- | --- | --- |
| **Gene** | **Probe Set** | **RefSeq ID** | **FC** | **pval** | **FC** | **pval** |
| Adam10 | 1450105_at | NM_007399 | 1.32 | 0.053 | 1.32 | 0.013 |
| Angpt1 | 1439066_at | NM_009640 | 1.52 | 0.135 | 2.48 | 0.011 |
| Cadm1 | 1417378_at | NM_001025600 | 1.38 | 0.015 | 1.27 | 0.004 |
| Col6a1 | 1448590_at | NM_009933 | 2.49 | 0.026 | 1.96 | 0.009 |
| Col6a2 | 1452250_a_at | NM_146007 | 2.11 | 0.061 | 1.84 | 0.015 |
| Ctnnb1 | 1420811_a_at | NM_001165902 | 1.32 | 0.019 | 1.21 | 0.001 |
| Dpysl3 | 1454613_at | NM_001136086 | 2.12 | 0.004 | 1.45 | 0.003 |
| Dpysl5 | 1449290_at | NM_023047 | 2.20 | 0.097 | 1.89 | 0.043 |
| Efnb3 | 1423085_at | NM_007911 | 2.69 | 0.002 | 1.69 | 0.003 |
| Fgfbp3 | 1453006_at | NM_028263 | 2.36 | 0.004 | 1.25 | 0.096 |
| Igf1r | 1426565_at | NM_010513 | 1.20 | 0.312 | 1.73 | 0.001 |
| Parvb | 1422060_at | NM_133167 | -1.32 | 0.932 | 3.46 | 0.001 |
| Sema5a | 1434776_at | NM_009154 | 3.23 | 0.028 | 1.63 | 0.025 |
| Sema6d | 1453055_at | NM_172537 | 1.84 | 0.004 | 1.71 | 0.001 |
| **Downregulated Genes** | | | **1228** | | **923** | |
| **Gene** | **Probe Set** | **RefSeq ID** | **FC** | **pval** | **FC** | **pval** |
| Cdh7 | 1444736_at | NM_172853 | -2.47 | 0.035 | -1.84 | 0.001 |
| Col19a1 | 1456953_at | NM_007733 | -1.99 | 0.002 | -1.56 | 0.002 |
| Epha3 | 1425575_at | NM_010140 | -3.01 | 0.002 | -1.61 | 0.056 |
| Epha4 | 1456863_at | NM_007936 | -1.72 | 0.019 | -1.42 | 0.010 |
| Fgf13 | 1418497_at | NM_010200 | -1.31 | 0.016 | -1.21 | 0.006 |
| Fgf14 | 1435747_at | NM_010201 | -1.36 | 0.018 | -1.79 | 0.001 |
| Parva | 1431375_s_at | NM_020606 | -1.64 | 0.032 | -1.14 | 0.147 |
| Shh | 1436869_at | NM_009170 | -1.27 | 0.075 | -1.21 | 0.099 |
| Sorbs2 | 1437197_at | NM_172752 | -1.55 | 0.001 | -1.34 | 0.005 |

**Table S6: Glioma invasion-related genes common with previously reported study by Kislin et al.** ^(reference 9 in the paper)^

| **Upregulated genes** | **Downregulated genes** |
| --- | --- |
| ACYP1 | ACYP2 |
| ATAD2 | ADAM23 |
| BUB1 (includes EG:100307076) | ADH4 (includes EG:127) |
| C11orf82 | APOE |
| CCNA2 | APOL2 |
| CCNB1 | ATP1B1 |
| CDC6 (includes EG:23834) | C4B (includes others) |
| CDK1 | CAMK2G |
| CDK12 | CD59 (includes EG:25407) |
| CENPA | CTSB |
| CENPE | DAZAP1 |
| CENPK | DTNA |
| CEP57 | DUSP22 |
| CEP57L1 | EIF4E3 |
| CKAP2L | FAM134A |
| CKS2 | FGF1 |
| CREB5 | GM2A |
| CSTF3 | GRAMD4 |
| DCAF17 | IGDCC4 |
| DENND2A | KCNB1 |
| DLGAP5 | LATS2 |
| DNTTIP2 | LOC283788 |
| DSEL | MAN2A2 |
| DTL | MAP7 |
| DTYMK | MB21D1 |
| ECT2 | MEG3 |
| FAM83D | MORF4L1 |
| FBXO5 | MORF4L2 |
| HMGB1 | MYBPC1 |
| HMGB2 | NGEF |
| HMMR | OPTN |
| HPS3 | PCSK1N |
| IRS1 | PGM2L1 |
| ITGB3BP | PHACTR2 |
| KIAA0101 | PLXNA4 |
| KIF18A | PRPS1 |
| KIF2C | RAB27A |
| KPNA2 | RAB6B |
| LMNB1 | RUFY3 |
| MAD2L1 | SERGEF |
| MATR3 | SIDT2 |
| MED28 | SIK1 |
| MELK | SPTBN1 |
| MND1 (includes EG:295160) | STOX1 |
| MYCBP | TRIM73/TRIM74 |
| NAA50 | UNC5B |
| NUF2 | ZNF540 |
| PBK |  |
| PCNA |  |
| PDLIM1 |  |
| PFDN4 |  |
| POC1A |  |
| POLH |  |
| PPAT |  |
| PRKAR2A |  |
| PRPF40A |  |
| PSMB2 |  |
| PTTG1 |  |
| PTX3 |  |
| QKI |  |
| QRSL1 |  |
| RBBP8 |  |
| RBMX |  |
| RPL15 |  |
| SET |  |
| SLC43A3 |  |
| SPATA6 |  |
| ST3GAL6 |  |
| TARDBP |  |
| TMEM60 |  |
| TMEM97 |  |
| TOP2A |  |
| TYMS |  |
| UBA2 |  |
| WDHD1 |  |
| WISP1 |  |
| ZNF207 |  |
| ZNF367 |  |
| ZNF627 |  |
| ZNF704 |  |

**References**

1. Clement-Ziza M, Munnich A, Lyonnet S, Jaubert F, Besmond C (2008) Stabilization of RNA during laser capture microdissection by performing experiments under argon atmosphere or using ethanol as a solvent in staining solutions. RNA 14: 2698-2704.

2. Ekins S, Nikolsky Y, Bugrim A, Kirillov E, Nikolskaya T (2007) Pathway mapping tools for analysis of high content data. Methods Mol Biol 356: 319-350.

3. Deribe YL, Wild P, Chandrashaker A, Curak J, Schmidt MH, et al. (2009) Regulation of epidermal growth factor receptor trafficking by lysine deacetylase HDAC6. Sci Signal 2: ra84.

4. Zimmer G, Rudolph J, Landmann J, Gerstmann K, Steinecke A, et al. (2011) Bidirectional ephrinB3/EphA4 signaling mediates the segregation of medial ganglionic eminence- and preoptic area-derived interneurons in the deep and superficial migratory stream. J Neurosci 31: 18364-18380.

5. Artigiani S, Conrotto P, Fazzari P, Gilestro GF, Barberis D, et al. (2004) Plexin-B3 is a functional receptor for semaphorin 5A. EMBO Rep 5: 710-714.

6. Li X, Law JW, Lee AY (2012) Semaphorin 5A and plexin-B3 regulate human glioma cell motility and morphology through Rac1 and the actin cytoskeleton. Oncogene 31: 595-610.

7. Li X, Lee AY (2010) Semaphorin 5A and plexin-B3 inhibit human glioma cell motility through RhoGDIalpha-mediated inactivation of Rac1 GTPase. J Biol Chem 285: 32436-32445.

8. Pan GQ, Ren HZ, Zhang SF, Wang XM, Wen JF (2009) Expression of semaphorin 5A and its receptor plexin B3 contributes to invasion and metastasis of gastric carcinoma. World J Gastroenterol 15: 2800-2804.

9. Sadanandam A, Rosenbaugh EG, Singh S, Varney M, Singh RK (2010) Semaphorin 5A promotes angiogenesis by increasing endothelial cell proliferation, migration, and decreasing apoptosis. Microvasc Res 79: 1-9.

10. Osterfield M, Egelund R, Young LM, Flanagan JG (2008) Interaction of amyloid precursor protein with contactins and NgCAM in the retinotectal system. Development 135: 1189-1199.

11. McCoy E, Sontheimer H (2007) Expression and function of water channels (aquaporins) in migrating malignant astrocytes. Glia 55: 1034-1043.

12. Neely JD, Amiry-Moghaddam M, Ottersen OP, Froehner SC, Agre P, et al. (2001) Syntrophin-dependent expression and localization of Aquaporin-4 water channel protein. Proc Natl Acad Sci U S A 98: 14108-14113.

13. Noell S, Ritz R, Wolburg-Buchholz K, Wolburg H, Fallier-Becker P (2012) An allograft glioma model reveals the dependence of aquaporin-4 expression on the brain microenvironment. PLoS One 7: e36555.

14. Warth A, Kroger S, Wolburg H (2004) Redistribution of aquaporin-4 in human glioblastoma correlates with loss of agrin immunoreactivity from brain capillary basal laminae. Acta Neuropathol 107: 311-318.

15. Kos FJ, Chin CS (2002) Costimulation of T cell receptor-triggered IL-2 production by Jurkat T cells via fibroblast growth factor receptor 1 upon its engagement by CD56. Immunol Cell Biol 80: 364-369.

16. Toyofuku T, Zhang H, Kumanogoh A, Takegahara N, Suto F, et al. (2004) Dual roles of Sema6D in cardiac morphogenesis through region-specific association of its receptor, Plexin-A1, with off-track and vascular endothelial growth factor receptor type 2. Genes Dev 18: 435-447.

17. Bowe MA, Mendis DB, Fallon JR (2000) The small leucine-rich repeat proteoglycan biglycan binds to alpha-dystroglycan and is upregulated in dystrophic muscle. J Cell Biol 148: 801-810.

18. Recktenwald CV, Leisz S, Steven A, Mimura K, Muller A, et al. (2012) HER-2/neu-mediated down-regulation of biglycan associated with altered growth properties. J Biol Chem 287: 24320-24329.

19. Clasper S, Vekemans S, Fiore M, Plebanski M, Wordsworth P, et al. (1999) Inducible expression of the cell surface heparan sulfate proteoglycan syndecan-2 (fibroglycan) on human activated macrophages can regulate fibroblast growth factor action. J Biol Chem 274: 24113-24123.

20. Florio P, Gazzolo D, Luisi S, Petraglia F (2007) Activin A in brain injury. Adv Clin Chem 43: 117-130.

21. Tanaka M, Murakami K, Ozaki S, Imura Y, Tong XP, et al. (2010) DIP2 disco-interacting protein 2 homolog A (Drosophila) is a candidate receptor for follistatin-related protein/follistatin-like 1--analysis of their binding with TGF-beta superfamily proteins. FEBS J 277: 4278-4289.

22. Xu J, Qi X, Gong J, Yu M, Zhang F, et al. (2012) Fstl1 antagonizes BMP signaling and regulates ureter development. PLoS One 7: e32554.

23. Soussi-Yanicostas N, Hardelin JP, Arroyo-Jimenez MM, Ardouin O, Legouis R, et al. (1996) Initial characterization of anosmin-1, a putative extracellular matrix protein synthesized by definite neuronal cell populations in the central nervous system. J Cell Sci 109 ( Pt 7): 1749-1757.

24. Hudson ML, Kinnunen T, Cinar HN, Chisholm AD (2006) C. elegans Kallmann syndrome protein KAL-1 interacts with syndecan and glypican to regulate neuronal cell migrations. Dev Biol 294: 352-365.

25. Soba P, Eggert S, Wagner K, Zentgraf H, Siehl K, et al. (2005) Homo- and heterodimerization of APP family members promotes intercellular adhesion. EMBO J 24: 3624-3634.

26. Eshkar Sebban L, Ronen D, Levartovsky D, Elkayam O, Caspi D, et al. (2007) The involvement of CD44 and its novel ligand galectin-8 in apoptotic regulation of autoimmune inflammation. J Immunol 179: 1225-1235.

27. Le Grand F, Jones AE, Seale V, Scime A, Rudnicki MA (2009) Wnt7a activates the planar cell polarity pathway to drive the symmetric expansion of satellite stem cells. Cell Stem Cell 4: 535-547.

28. von Maltzahn J, Bentzinger CF, Rudnicki MA (2012) Wnt7a-Fzd7 signalling directly activates the Akt/mTOR anabolic growth pathway in skeletal muscle. Nat Cell Biol 14: 186-191.

29. Bentzinger CF, Wang YX, von Maltzahn J, Soleimani VD, Yin H, et al. (2013) Fibronectin regulates Wnt7a signaling and satellite cell expansion. Cell Stem Cell 12: 75-87.

30. Tuli A, Sharma M, McIlhaney MM, Talmadge JE, Naslavsky N, et al. (2008) Amyloid precursor-like protein 2 increases the endocytosis, instability, and turnover of the H2-K(d) MHC class I molecule. J Immunol 181: 1978-1987.

31. Morris CR, Petersen JL, Vargas SE, Turnquist HR, McIlhaney MM, et al. (2003) The amyloid precursor-like protein 2 and the adenoviral E3/19K protein both bind to a conformational site on H-2Kd and regulate H-2Kd expression. J Biol Chem 278: 12618-12623.
